# Supplementary material for: Elucidation of the role of metals in the adsorption and photodegradation of herbicides by metal-organic frameworks
Source: Nat Commun. 2024 Feb 17;15:1459. doi: 10.1038/s41467-024-45546-y (PMC10874385; doi:10.1038/s41467-024-45546-y)
Supplement: Supplementary file 1 — Supplementary Information [file 41467_2024_45546_MOESM1_ESM.pdf]

Supplementary Information

Elucidation of the role of metals in the adsorption and photodegradation of herbicides by metal-organic frameworks

Nan Chieh Chiu,[1†] Jacob M. Lessard,[1†] Emmanuel Nyela Musa,[1†] Logan S. Lancaster,[2] Clara Wheeler,[2] Taylor D. Krueger,[2] Cheng Chen,[2] Trenton C. Gallagher,[2] Makenzie T. Nord,[2] Hongliang Huang,[3*] Paul Ha-Yeon Cheong,[2*] Chong Fang,[2*] Kyriakos C. Stylianou[1*]

[1] Materials Discovery Laboratory (MaD Lab), Department of Chemistry, Oregon State University, 153 Gilbert Hall, Corvallis, Oregon 97331, United States

[2] Department of Chemistry, Oregon State University, Corvallis, 153 Gilbert Hall, Oregon 97331, United States

[3] State Key Laboratory of Separation Membranes and Membrane Processes, School of Chemical Engineering and Technology, Tiangong University, Tianjin 300387, China

†Denotes equal contribution

Emails: huanghongliang@tiangong.edu.cn, cheongh@oregonstate.edu, chong.fang@oregonstate.edu, and kyriakos.stylianou@oregonstate.edu

Supplementary methods

The powder X-ray diffraction (PXRD) was conducted on a Rigaku MiniFlex equipped with a monochromated Cu Kα radiation (λ= 1.5418 Å) at 40 kV and 25 mA with a scan speed of 0.2 s per step, a step size of 0.02° in 2θ and a 2θ range of 3–30° at ambient temperature.

Nitrogen adsorption-desorption isotherms were collected at 77 K and 1 bar using a Micromeritics 3FLEX apparatus. Before data collection, samples were degassed at 130 °C for 12 h under a vacuum to remove residual solvent molecules and moisture within the structure. The Brunauer-Emmett-Teller (BET) surface areas were estimated from the amount of N_2 adsorbed via the BET equilibrium equation.

UV-visible absorbance was obtained using a PerkinElmer UV-visible spectrometer equipped with an integrating sphere. The diffuse reflectance was collected by depositing the powders within the solid sample holder equipped with a quartz window and the Kubelka-Munk (K-M) function was applied to the raw spectral data.

Electrical Impedance Spectroscopy (EIS) was conducted using a Swagelok cell configuration in a VMP-3 multichannel workstation. Cells were composed of a working electrode, a filter paper separator, an Ag/AgCl reference electrode, and an activated carbon counter electrode. Working electrodes were composed of 10 wt. % ketjenblack (KB) carbon, 10 wt. % polyvinylidene fluoride as binder, and 80 wt. % active material. Tests were conducted in a frequency range of 200 kHz to 50 MHz for an amplitude of 10 mV and using a 0.1 m Na_2SO_4 as electrolyte.

Chemical stability measurement. Chemical stability experiments were carried out by centrifuging the photocatalytic solution and collecting the solid material, which was then dried in the oven at 70 °C overnight and analyzed by PXRD.

Thermogravimetric analyses (TGA) were carried out with a standard Thermogravimetry/Differential Thermal Analysis (TG-DTA) analyzer within the temperature range 30–650 °C with a heating rate of 10 °C/min under air flow (100 mL/min).

X-ray photoelectron spectroscopy (XPS). For X-ray photoelectron spectroscopy (XPS), a copper double-sided tape (TED PELLA, INC.) was placed on a standard stainless-steel SPECS holder. The MOF powder was pressed on the tape and loaded to the ultrahigh vacuum (UHV) system through a fast entry lock (base pressure ∼1×10^{-8} mbar). X-ray photoelectron spectroscopy (XPS)

was performed in a custom-built SPECS Surface Nano Analysis GmbH near-ambient pressure (NAP) system. The system has an ultrahigh UHV manipulator for standard XPS in UHV (< 2×10^{-10} mbar) and a near-ambient pressure (NAP) cell for NAP-XPS, which can be transferred into the analysis chamber. The NAP cell can be pressurized up to ∼10 mbar while maintaining a vacuum of 1×10^{-9} mbar in the surrounding chamber. XPS experiments were measured on as-loaded samples at room temperature. The valence band value (or valence band maximum) of all MOFs was determined by using a linear extrapolation method. By linear fitting the leading edge of the valence band and linearly fitting the flat energy distribution, the intersection of these two lines is the experimental VBM value (Supplementary Figure 4).[1,2]

Electrochemical measurement. The photoelectrochemical performances of the MOF samples were measured by a CHI760E Electrochemical Workstation (Shanghai Chenhua Instrument Co., Ltd., Shanghai, China). The Pt foil electrode, the Ag/AgCl electrode, and the as-prepared MOF catalysts were used as the counter, the reference, and the working electrodes, respectively. A 0.5 mol L^{-1} Na_2SO_4 solution was employed as the electrolyte. Mott−Schottky (MS) plots were performed at the frequencies of 500, 1000, and 1500 Hz. To convert the obtained flat band potential vs. Ag/AgCl to it vs. RHE (NHE at pH=0), the following equation is used: $E_{RHE} = E_{Ag/AgCl} + 0.059\, pH + E^0_{Ag/AgCl}$, where E_{RHE} is the converted potential vs. RHE, $E^0_{Ag/AgCl}$ = 0.1976 V at 25 °C, and $E_{Ag/AgCl}$ is the experimentally measured potential against Ag/AgCl reference.

Femtosecond transient absorption (Fs-TA) measurements. Fs-TA experiments were performed on a home-built ultrafast laser spectroscopic setup using a mode-locked Ti:sapphire oscillator (Mantis-5, Coherent, Inc.) as the seed for the regenerative amplifier (Legend Elite-USP-1K-HE, Coherent, Inc.) to produce a 800 nm pulse train with ~35 fs pulse duration, 1 kHz repetition rate, and ~3.6 W average power.[3,4] The 400 nm actinic pump was generated from a fraction of the fundamental beam directed through a 1.0-mm-thick type-I BBO crystal to generate 400 nm pulses via second harmonic generation (SHG). A λ/2 waveplate and polarizer were placed in the pump beamline, allowing the polarization to be set to the magic angle (54.7°) relative to the probe pulse to avoid anisotropic effects. The actinic pump (~0.25 mW average power) was then aligned onto a quadruple-pass translation stage controlled by a stepper-motor (NRT-150, Thorlabs, Inc.) which covers a time delay up to ~3.6 ns.

The probe was generated by directing a small portion of the fundamental laser output through a 2-mm-thick quartz cuvette filled with deionized water. Both the pump and probe pulses were focused onto a 1-mm-pathlength quartz cuvette containing the sample. Afterwards, the pump was blocked using a pinhole while the probe was directed into a spectrograph (IsoPlane SCT-320, Princeton Instruments, Inc.) and dispersed inside using a reflective grating (300 grooves/nm, 300 nm blaze wavelength) onto a CCD array camera (PIXIS:100F, Princeton Instruments, Inc.). The signal was synchronized with a phase-locked optical chopper (New Focus 3501) placed in the pump beam path, set to 500 Hz. To increase the signal-to-noise ratio, all the spectral data were collected using 3000 shots per point and a total of five sets, thus each time point is the average from 7500 spectra.

MOF samples were suspended in DMF and sonicated for 30 minutes after which the suspension were allowed to settle for 30 minutes to avoid precipitation during experiments.[5] The suspensions were then diluted with DMF as needed to reach ~0.3–0.5 OD per mm. Fe-TBAPy was substantially less stable in suspension and only achieved an OD of ~ 0.1 per mm. The ligand samples were prepared in the same manner in both DMF and ACN solvents. During the experiments, a miniscule stir bar was used to continuously stir the sample. The UV-vis spectrum of each sample was taken before and after each fs-TA experiment to ensure that no light-induced degradation was detected.

Fs-TA data analysis and further discussions. The steady-state and time-resolved electronic spectroscopy on these functional TBAPy-MOFs with various metal nodes (Figures 3 and S6-8) can provide deep mechanistic insights into their working mechanisms. As a foundation for this line of inquiry, steady-state electronic spectroscopy of the isolated ligand "linker" molecule, H_4TBAPy, in dimethylformamide (DMF, see Supplementary Figure 9a) shows a dominant near-UV absorption peak at 392 nm and an emission peak at 447 nm. In acetonitrile (ACN), H_4TBAPy is insoluble and forms suspensions, displaying two red-shifted absorption peaks at ~435 and 470 nm with a weak shoulder around 405 nm. Significant scattering from the suspended particles adds a long red tail to the absorption profile which may obscure some weak absorption features above 500 nm. The emission spectrum in ACN is similarly red-shifted and displays two peaks at ~497 and 525 nm. The spectral change is the result of aggregation of TBAPy molecules with increasing π-π interactions to allow for delocalized states with red-shifted emission.[6-11] Notably, the fluorescence intensity is decreased by more than an order of magnitude in ACN compared to DMF with a comparable optical density (OD, see Supplementary Figure 9a), indicating the greatly enhanced nonradiative decay within the aggregate.[6,7,12]

The Sc-TBAPy, Al-TBAPy, and Y-TBAPy MOFs all exhibit similar absorption profiles (Supplementary Figure 6a) to the solvated ligand with peak maxima at 386, 388, and 419 nm, respectively. The fluorescence spectra of all the MOFs (Supplementary Figure 6b) also resemble the solvated ligand, each featuring a dominant peak. However, compared to the ligand emission at 447 nm in DMF, Sc-TBAPy and Y-TBAPy MOFs display red-shifted emission peaks at 460 and 466 nm, whereas Al-TBAPy MOF displays a blue-shifted emission peak at 445 nm. In contrast, Fe-TBAPy MOF shows a very weak and broad absorption band around 500 nm and significantly weakened fluorescence compared with the other MOFs. The observed broadened emission bands of all MOFs versus the ligand in solution likely indicate the inhomogeneous broadening as a result of the solid-state MOF structure, along with potential contributions from additional emissive species such as excimers,[8,13,14] which could form in MOFs with densely packed linkers in highly ordered arrays. These emissive species may occur within the MOFs via interchromophoric interactions; however, the relatively small changes to the emission profiles (Supplementary Figure 6b inset) versus the observed change for H_4TBAPy upon suspension in ACN (Supplementary Figure 9a) indicates that these inter-linker states do not dominate the fluorescence behavior of MOFs.

By implementing femtosecond transient absorption (fs-TA) spectroscopy on the H_4TBAPy ligand in DMF, the data show a strong excited-state absorption (ESA) band at ~700 nm, a weaker ESA band at ~510 nm, and a negative feature below 500 nm (Supplementary Figure 9b). Upon suspension in ACN, the stimulated emission (SE) peak of H_4TBAPy red-shifts to 490 nm and a second, prominent SE band appears at 540 nm (Supplementary Figure 9d), closely matching the steady-state emission profile (Supplementary Figure 9a). The 700 nm ESA peak, which is visible at early time, decays largely within the first 25 picoseconds (ps). This shorter lifetime of the 700 nm ESA peak demonstrates the greater nonradiative quenching of the monomeric excitation due to interchromophoric interactions. Both SE peaks decay and undergo a slight redshift initially, then on the same 25 ps timescale, they exhibit a clear blueshift (i.e., from 493 to 478 nm, and 559 to 553 nm). The observed blueshift of the higher-energy SE band likely stems from an overlapping ground-state bleach (GSB) band (absorption peak at ~470 nm) which obfuscates a near-complete decay of the SE band. However, the blueshift of the 540 nm SE peak cannot be easily explained by GSB, instead it likely tracks the rise of an adjacent band at ~580–600 nm or a long-lived but weak feature in a similar region. Following the initial ~25 ps, the remaining weak ESA band is

broad and displays a positive shoulder below 600 nm, which can be attributed to delocalization of the exciton state.[15-17] Interchromophoric interactions provide additional relaxation pathways for the initially generated emissive state, and facilitate the formation of some low-energy absorption features below ~600 nm.[7,15,18]

After ~200 ps, a broad SE band rises to dominate the entire spectrum, and for the probe region above 600 nm the rise continues throughout the time window (3.6 ns). This result can be assigned to the formation of excimers between the pyrene cores of neighboring TBAPy linkers[14,15,18,19] which, due to symmetry requirements, must undergo conformational changes to reach an emissive excimer state.[14,20] Global analysis[21,22] of the TA data yields four time constants for H_4TBAPy in ACN: 900 fs, 12 ps, 300 ps, and 35 ns (Supplementary Figure 9d), which represent Franck-Condon relaxation, the monomer S_1 state lifetime, excimer formation, and the excimer fluorescence lifetime, respectively. We note that the largest time constant is considerably greater than the detection time window, yet it can serve as a good estimate of the lifetime as the exponential function dictates in a least-squares fit.[23] The lifetime has been reproducibly retrieved from our experiments and agrees well with reports of 10–100 ns lifetimes of pyrene-derived excimers.[14,17,24]

For comparison, fs-TA spectra of the MOFs suspended in DMF (Figure 3) exhibit clear ligand-derived features including an ESA band around 700 nm, but a second maximum is observed around 650 nm for all the MOFs except Fe case, which only shows a weak and broad ESA band above ~600 nm (Figure 3c). This second ESA peak represents a lower energy state facilitated by the MOF-framework and may be the result of some delocalization of the molecular exciton between linkers. The spectra of Al-TBAPy, Fe-TBAPy, and Y-TBAPy display ~540 nm SE bands (Figures 3b-d), almost identical to that observed for the suspended ligand in ACN **(**Supplementary Figure 9d**)**, which can be attributed to interchromophoric interactions analogous to the aggregate. The presence of these features around time zero infers that the linkers are in association before photoexcitation. On the other hand, Sc-TBAPy (Figure 3a) profoundly resembles the solvated ligand in DMF (Supplementary Figure 9b) with negative features only below ~500 nm and a strong ESA around 700 nm, albeit with a second peak around 650 nm. As mentioned above, the ligand in DMF shows a strong ESA band at ~700 nm, a weaker ESA band at ~510 nm, and a negative feature below 500 nm. This negative feature can be assigned to SE as it falls in steady-state emission region and there is no clear ground-state absorbance of the ligand above 450 nm (Supplementary Figure 9a).

In order to gain a clearer picture of the altered excited states, fs-TA spectra were collected in the visible region[21,25-27] for each MOF (Figure 3) and compared with the "pure" ligand in DMF and ACN (Figures S9b, d). In particular for the "solvated" DMF case, the 700 and 510 nm ESA peaks are both present from time zero and correspond to transitions from S_1 to higher-lying electronic states.[14,15,28-30] Over the first few ps, the SE peak and 700 nm ESA peak both red-shift and rise while the ESA band between 500 and 650 nm decays. The simultaneous redshift of ESA and SE peaks reflects a reduction in both $S_1 \rightarrow S_n$ and $S_1 \rightarrow S_0$ transition energies, strongly hinting the involvement of conformational changes. The TA signal does not decay entirely within 3.6 ns, but the SE is absent after ~3 ns while a third ESA feature at ~550 nm becomes increasingly apparent as it exhibits a longer lifetime than the rest of the broad ESA band (see the asterisk in Supplementary Figure 9b). The rise of this 550 nm band is obscured by the prominent neighboring ESA bands which decay on similar timescales; however, the signature of this band is visible after ~5 ps, indicating a formation time of a few ps at most. Similar spectral features have been attributed to the product of charge transfer (CT) involving the pyrene core,[19,30,31] therefore, this feature could represent a semi-trapped population (potentially a twisted intramolecular charge transfer (TICT) state),[32,33] intermolecular charge/electron transfer, or a weakly allowed transition from the CT state.

Since decay dynamics retrieved for the 700 and 550 nm ESA bands diverge both at early and late time points (Supplementary Figure 9c), these bands can be confidently assigned to different electronic states.[12,34] The ultrafast timescale is discordant with intermolecular charge transfer between TBAPy monomers which is diffusion-controlled and should occur on the sub-ns timescale.[12,35,36] This leaves the most likely origin of the 550 nm ESA band as a small, semi-trapped, intramolecular CT population which undergoes a delayed relaxation to the ground state, corroborating key discussions on the ultrafast CT dynamics in M^{3+}-TBAPy MOFs (see Figure 3 in main text).

Global analysis of fs-TA data on MOFs. Global analysis of the fs-TA spectra provides the evolution- and decay-associated difference spectra (EADS and DADS) of Sc-TBAPy and Al-TBAPy (Supplementary Figure 7) as well as Fe-TBAPy and Y-TBAPy (Supplementary Figure 8) with similar early-time dynamics in the first two MOFs but more prominent SE dynamics in the latter two MOFs. For Sc-TBAPy case the signal is well fit by four lifetimes: 400 fs, 3.5 ps, 45 ps,

and 1.5 ns (Figures S7a). There is a small apparent rise associated with the sub-ps time constant which implies a rise of a relatively weak ESA band or a rapid decay of the aggregate SE in the same region (see the cyan shade in Figures S7a,c**).** Following the fast rise of the ~550 nm ESA peak, it displays a notably longer lifetime than the rest of ESA band and becomes the dominant species with the longest 1.5 ns lifetime (green curve, Supplementary Figure 7a). For Al-TBAPy, it requires five components for the best fit: 600 fs, 5, 47, 750 ps, and 2.3 ns (Supplementary Figure 7b). The first time constant can be attributed to ultrafast CT, followed by two-time constants on stabilization and relaxation of the excited TBAPy linker,[14,29] and two longer time components representing the formation and emission of excimers. The initial time constants in the Al case are all lengthened versus the Sc case, hinting a prolonged emissive state that accounts for the enhanced fluorescence of Al-TBAPy (Supplementary Figure 6b). The additional time constants in Al-TBAPy correspond to the electronic dynamics of nascent excimers (Supplementary Figures 7b,d), which cannot be retrieved for Sc-TBAPy since no observable SE band is formed on the ps-to-ns timescales (Supplementary Figure 7a).

Photocatalytic reactions and sample analysis

Characterization of oxidation products. Three resonance active nuclei in GP (^{1}H, ^{13}C, and ^{31}P) were analyzed conventionally using solution one-dimensional nuclear magnetic resonance (1D NMR) spectroscopy for the identification and assignment of peaks to products of GP degradation. Prior to the NMR analysis, the GP solution containing the MOF was first centrifuged, and the clear solution was then collected. Sodium 3-(trimethylsilyl)-1-propane sulfonate (DSS) was used as standard reference material for the ^{1}H spectra. The reference spectra of the standards: GP, AMPA, glycine, formic acid, formaldehyde, methanol, acetic acid, and phosphoric acid were also collected and analyzed for the assignment of degradation products. The ^{1}H, ^{13}C, and ^{31}P NMR for all the pure standards were obtained, followed by the collection of intermediate products from the degradation of GP. The water suppression pulse program, zero gain excitation sculpting (zgesp) in the Bruker library was executed on the strong water signal observed in the ^{1}H NMR spectra of the photodegradation samples at δ=4.7 (s).

Percent conversion of GP and degradation products. The relative concentration of glyphosate and all degradation products present in the solution were calculated from the concentration normalized reference DSS resonance peak at 0 ppm. All ^{1}H peaks corresponding to DSS, GP and

other products were integrated, and the relative concentrations were calculated using the relative conversion approach shown below:[37,38]

$$M_x = \left(\frac{I_x}{I_{DSS}}\right)\left(\frac{N_{DSS}}{N_x}\right)(M_{DSS}) \quad (1)$$

where M_x is the concentration in (mol/L) of GP, or any degradation product, M_{DSS} is the concentration of the DSS reference solution (1.00×10^{-4} M), I_x is the sum of the integration values for the observed ^{1}H NMR peak for GP or other degradation products, N_{DSS} is the number of nuclei giving rise to the DSS peak ($N_{DSS} = 9$), and N_x as the number of nuclei giving rise to GP ($N_x =$ 4), glycine ($N_x = 2$), formic acid ($N_x = 1$), acetic acid ($N_x = 3$), and AMPA ($N_x = 3$) peaks. The relative percent conversion for glyphosate and all degradation products were calculated using the equation below.

For example, the percent conversion to glycine is shown below:

$$\%\ conversion_{\boldsymbol{Glycine}} = \left[\frac{M_{Glycine}}{(M_{GP}+M_{AMPA}+M_{Formic\ acid}+M_{Glycine})}\right] \times 100\% \quad (2)$$

Inductively Coupled Plasma Optical Emission spectroscopy (ICP-OES) analysis. Concentrations of Sc^{3+} were analyzed on a Spectro Arcos II ICP-OES operated in end-on (axial) configuration. Matrix matched standards were prepared utilizing ultrapure reagents and single element standard solutions provided by Inorganic Ventures. Standards solutions were prepared by diluting by volume using ultrapure 2% HNO_3 for different concentrations (0, 50, 100, 500, 750 ppb). Internal errors for all data (standards and samples) were generated as three runs of 28-second integrations each. Supplementary Table 1 shows the analytical line, limit of detection, and maximum calibrated range.

Sample is prepared by adding 10 mg Sc-TBAPy MOF in 3mL 1.5mM GP solution and irradiated by 300-watt Xe lamp with 360 nm cut-off filter for 8hr. Solution was filtered with 0.22 µm syringe filter. The filtered solution was then diluted 2.5-fold by volume using ultrapure 2% HNO_3 (total sample volume was 5 ml).

Supplementary Table 1. ICP-OES measurement parameters.

Element	Analytical Line	LOD	Maximum calibrated range
Scandium	424.683 nm	0.76 ppb	750 ppb

Computational method. For energy calculations, each MOF was optimized starting from the In-TBAPy structure derived from the .cif file of the crystal structure of Stylianou et al.[20] The metal was swapped out for Sc, Y, Fe, and Al and relaxed in VASP[39-42] using PBE and a plane-wave basis set with a C_{mmm} kpoint mesh grid and a kinetic energy cutoff of 400 eV. We used Gaussian smearing for partial occupancies in each orbital with a width of 0.03 eV and a blocked Davidson iteration scheme for the electronic minimization algorithm. The calculation of density of states (DOS) included no ionic updates and ranged from –13 to 7 eV with 1500 intervals, though only –4 to 4 eV is shown.

DFT is known to underestimate bandgaps, while we were unable to use hybrid functionals due to the computational cost resulting from the size of the unit cell. The open *d* shell introduced the likelihood of highly correlated *d* electrons. To mitigate the effects on the band gap of the highly correlated *d* electrons of the open-shell Fe^{3+}, we used Dudarev *et al.*'s rotationally invariant Hubbard corrections.[43] These procedures were also used with the other three metals in order to maintain consistency across the calculations. The values for U were determined on the basis of similarity of the calculated bandgap to experimental results and were found for hydrogen, carbon, oxygen, and the metal to be 0, 7, 5, and 5 eV, respectively.

Partial charge densities from VASP output were taken at the valence band maximum and the conduction band minimum, and visualized as isodensity surfaces (isodensity level of 0.000308797) in VESTA using VASPKIT[44] as a file converter.

Binding Energies. DFT calculations were carried out using the CP2K code.[45] All calculations employed a mixed Gaussian and planewave basis sets. Core electrons were represented with norm conserving Godedecker -Teter-Hutter pseudopotentials,[46-48] and the valence electron wavefunction was expanded in a double-zeta basis set with polarization functions[49] along with an auxiliary plane correlation functional of Perdew, Burke, and Enzerhof of (PBE)[50] being used. Each configuration

was optimized with the Broyden-Fletcher-Goldfarb-Shanno (BFGS) algorithm with SCF convergence criteria of 1.0×10^{-6} au. To compensate for the long-range van der Waals dispersion interaction between the adsorbate and the MOF skeleton, the DFT-D3 scheme[51] with an empirical damped potential calculation was implemented. The interaction energy of GP moiety on M^{3+}-TBAPy was calculated as follows (Supplementary Table 2):

$$\Delta E = E(M^{3+}\text{-TBAPy} - GP) - E(M^{3+}\text{-TBAPy}) - E(GP) \quad (3)$$

where $E(M^{3+}\text{-TBAPy} - GP)$ is the total energy of the M^{3+}-TBAPy with the bound GP moiety, $E(M^{3+}\text{-TBAPy})$ is the total energy of the MOF itself, and E(GP) is the energy of the GP moiety.

Supplementary Figures and Tables

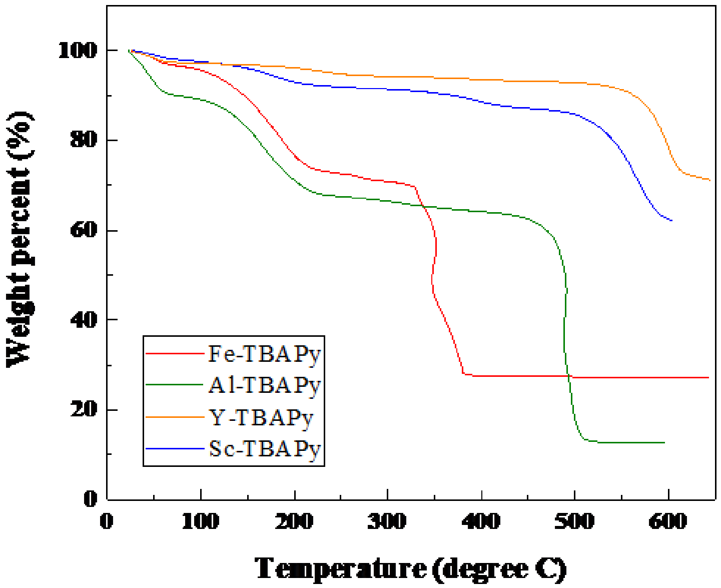

Supplementary Figure 1. Thermogravimetric analysis of Sc-TBAPy (blue), Al-TBAPy (green), Y-TBAPy (yellow), and Fe-TBAPy (red).

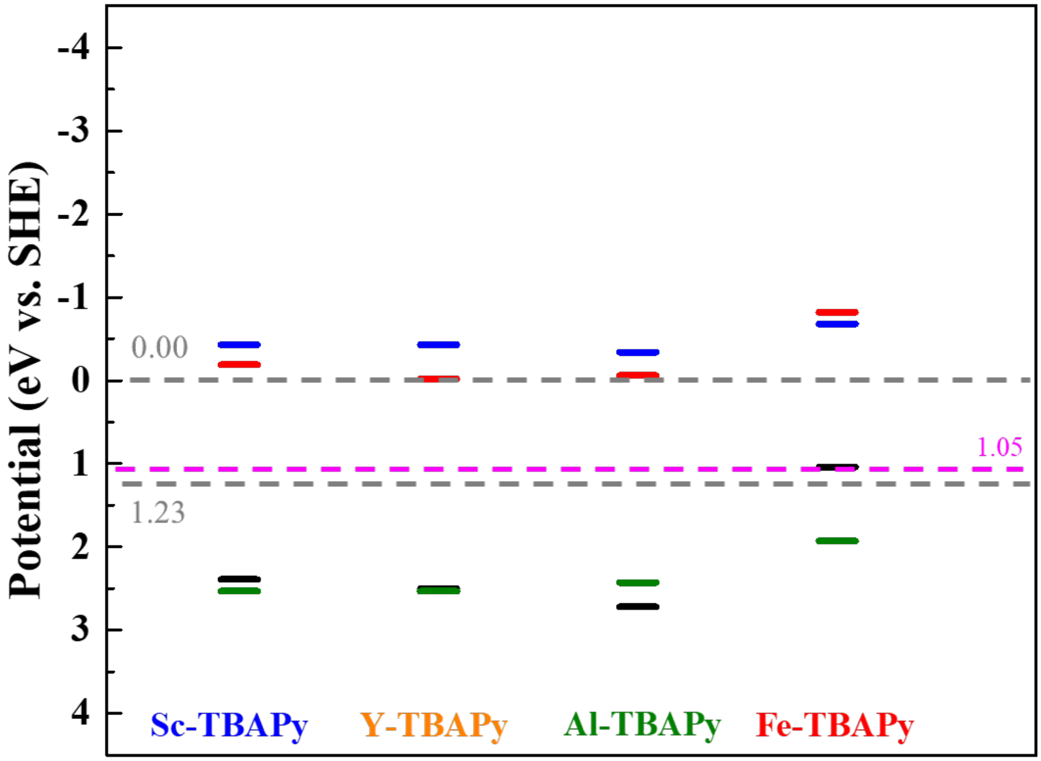

Supplementary Figure 2. Band gap diagram based on experimental data and DFT calculations. Experimental conduction band (CB, red) and valence band (VB, black) values for Sc-TBAPy, Y-TBAPy, Al-TBAPy, and Fe-TBAPy are represented in the diagram. Computational CB (blue) and VB (green), as well as HOMO values for glyphosate (pink dashed line)[52], are shown. H^+/H_2, 0.00 eV vs. SHE (standard hydrogen electrode); O^{2-}/O_2, 1.23 eV vs. SHE (gray dashed lines).

Supplementary Table 2. List of energy band values of the experimental conduction band (CB) and valence band (VB) values for Sc-TBAPy, Y-TBAPy, Al-TBAPy, and Fe-TBAPy, as well as the computational HOMO and LUMO values of glyphosate (GP).[52]

	Valence band (eV, vs. SHE)	Conduction band (eV, vs. SHE)	Bandgap (eV)
Sc-TBAPy	2.39	– 0.19	2.58
Y-TBAPy	2.50	– 0.02	2.52
Al-TBAPy	2.72	– 0.06	2.78
Fe-TBAPy	1.04	– 0.82	1.86
Glyphosate	1.05	– 3.17	4.22

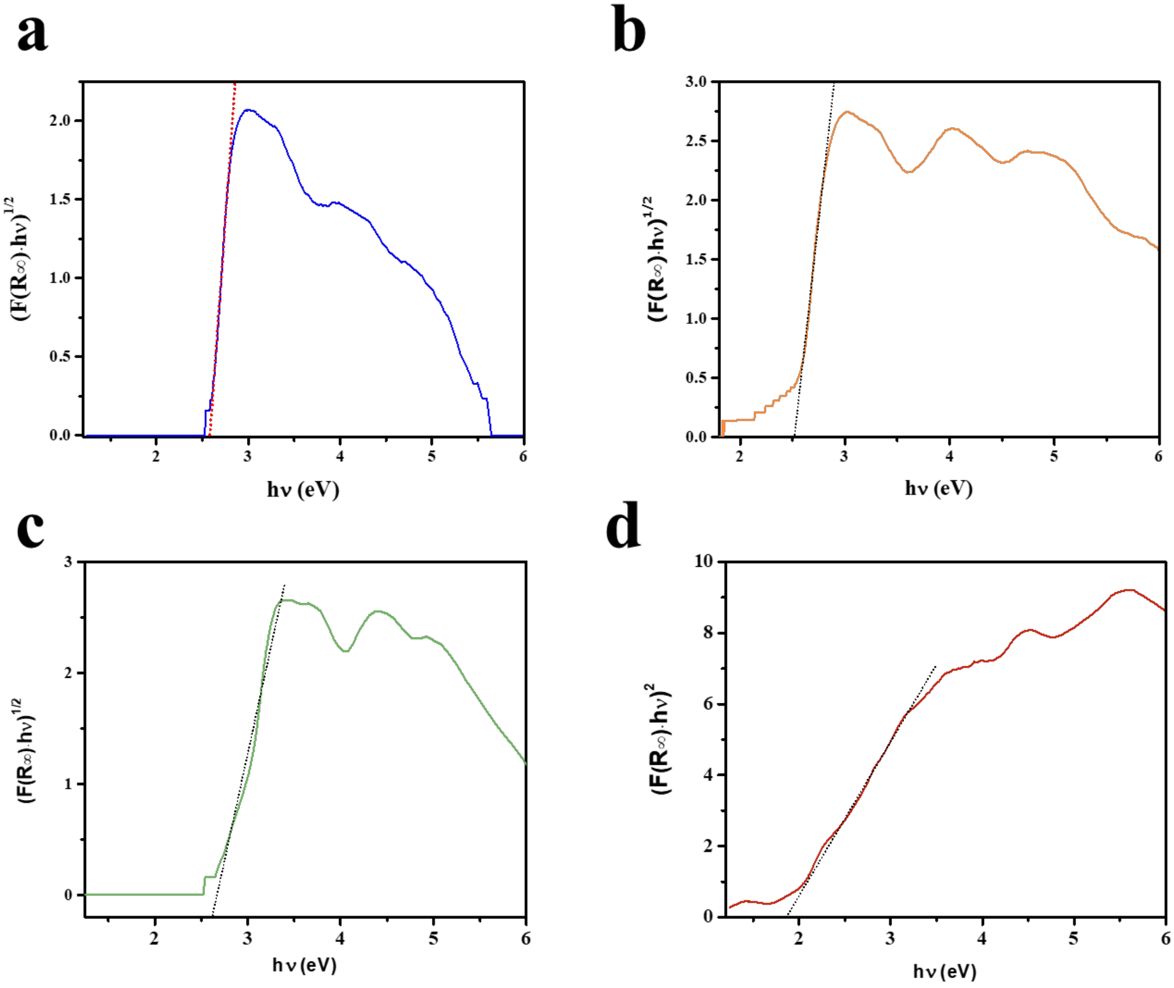

Supplementary Figure 3. Tauc plots of **a.** Sc-TBAPy, **b.** Y-TBAPy, **c.** Al-TBAPy, **d.** Fe-TBAPy data shown in Figure 2d.

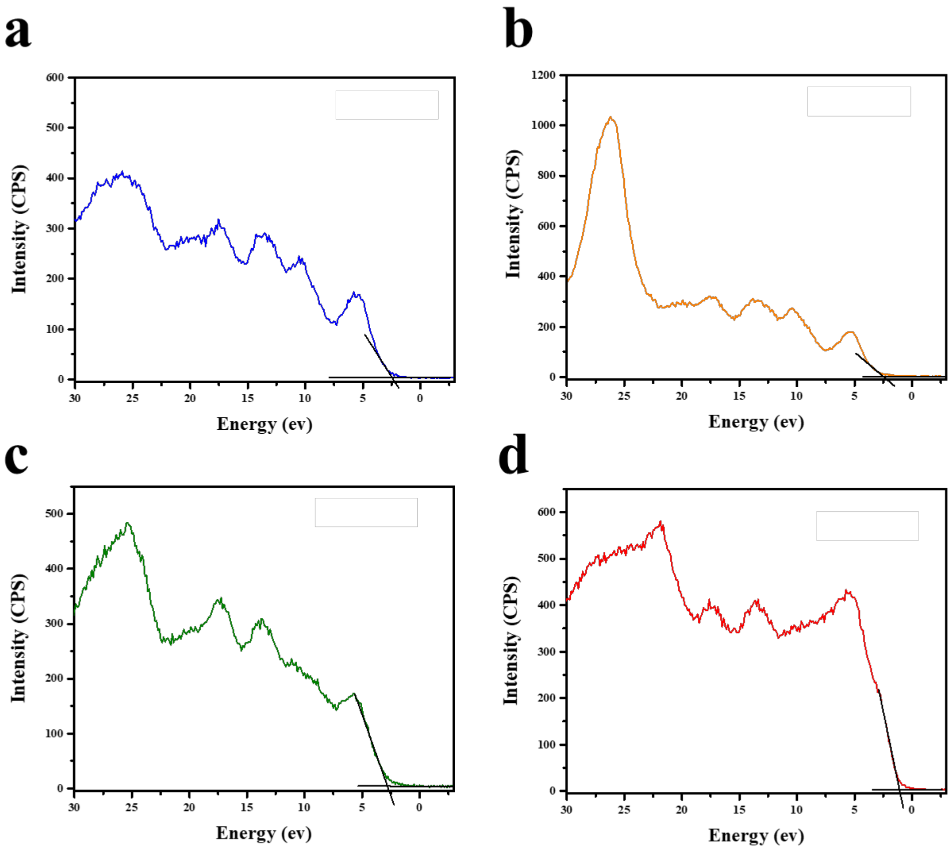

Supplementary Figure 4. XPS spectra of **a.** Sc-TBAPy, **b.** Y-TBAPy, **c.** Al-TBAPy, **d.** Fe-TBAPy. The valence band levels of all MOFs shown in Supplementary Table 1 were based on the fitting of the XPS spectra.

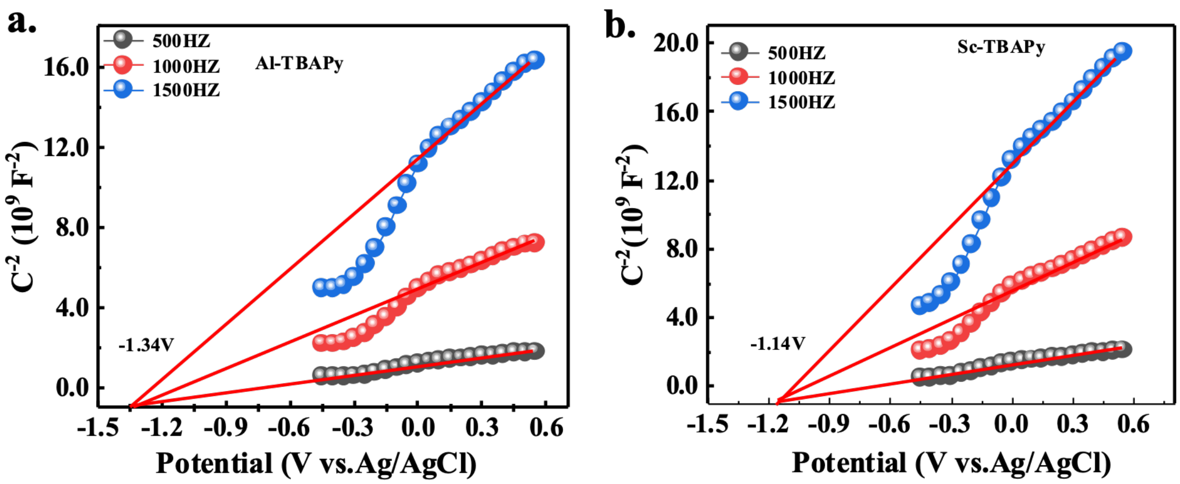

Supplementary Figure 5. Mott–Schottky plots for Al-TBAPy and Sc-TBAPy. Measurements were collected at 500, 1000, and 1500 HZ. The CB energy levels for Al-TBAPy and Sc-TBAPy were calculated to be at –0.8 V and –0.6 V, respectively.[53]

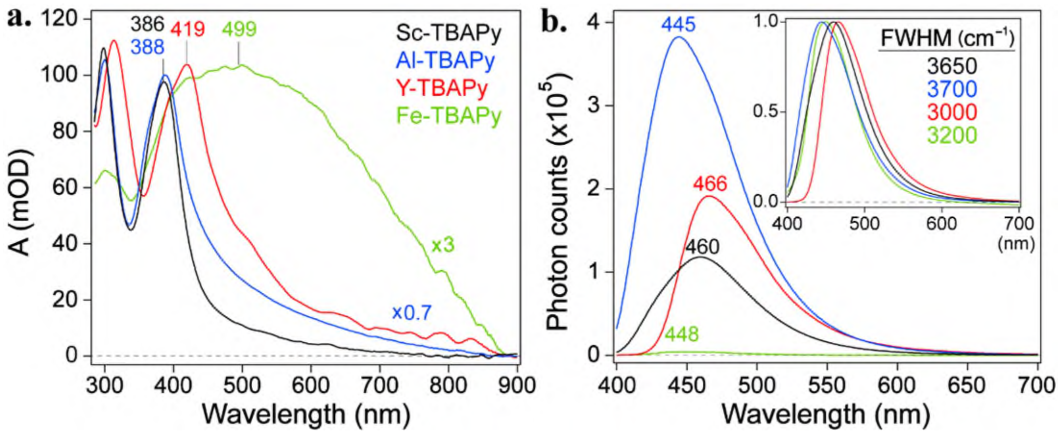

Supplementary Figure 6. Steady-state electronic spectra of TBAPy-based MOFs suspended in DMF. **a.** UV-visible absorption spectra of Sc-TBAPy (black), Al-TBAPy (blue), Y-TBAPy (red), and Fe-TBAPy (green) with the peak wavelengths (nm unit) denoted. The scaling factors for the Al and Fe spectra are color-coded and listed. **b.** Fluorescence spectrum of each MOF upon 385 nm excitation. Peak emission wavelengths (in nm unit) are labeled. The comparison of the normalized spectra and peak full width at half maximum (FWHM) is shown in the inset.

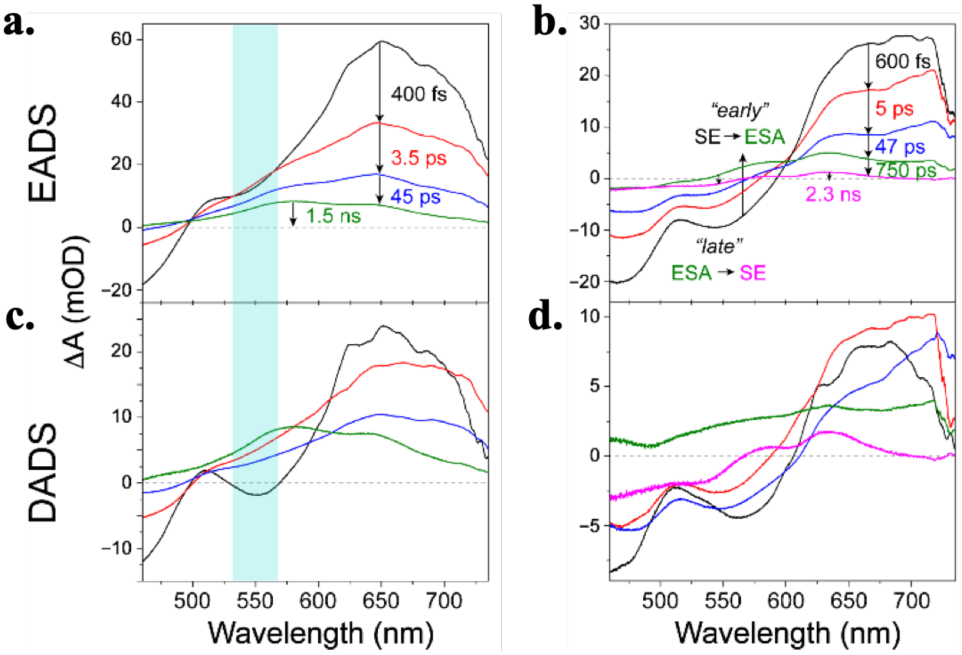

Supplementary Figure 7. Global analysis of fs-TA spectra of Sc-TBAPy and Al-TBAPy suspended in DMF after 400 nm excitation. The evolution-associated difference spectra (EADS) for Sc-TBAPy and Al-TBAPy are respectively shown in **a.** and **b.** with a sequential model, while the corresponding decay-associated difference spectra (DADS) with a parallel model are respectively shown in **c.** and **d.** The lifetimes associated with each species are color-coded (black→red→blue→green→pink) in the EADS, using vertical arrows to emphasize the spectral progression over time.

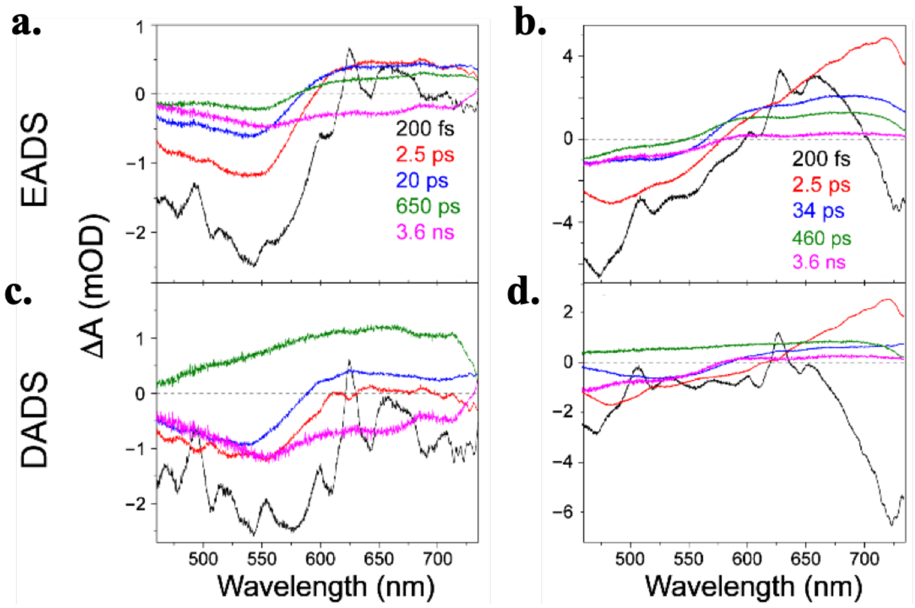

Supplementary Figure 8. Global analysis of fs-TA spectra of Fe-TBAPy and Y-TBAPy suspended in DMF. The evolution-associated difference spectra (EADS) for Fe-TBAPy and Y-TBAPy are respectively shown in **a.** and **b.** with a sequential model, while the corresponding decay-associated difference spectra (DADS) with a parallel model are respectively shown in **c.** and **d.** The lifetimes associated with each species are color-coded (black→red→blue→green→pink) in the EADS. The green traces below ~575 nm show a sign reversal between EADS and DADS.

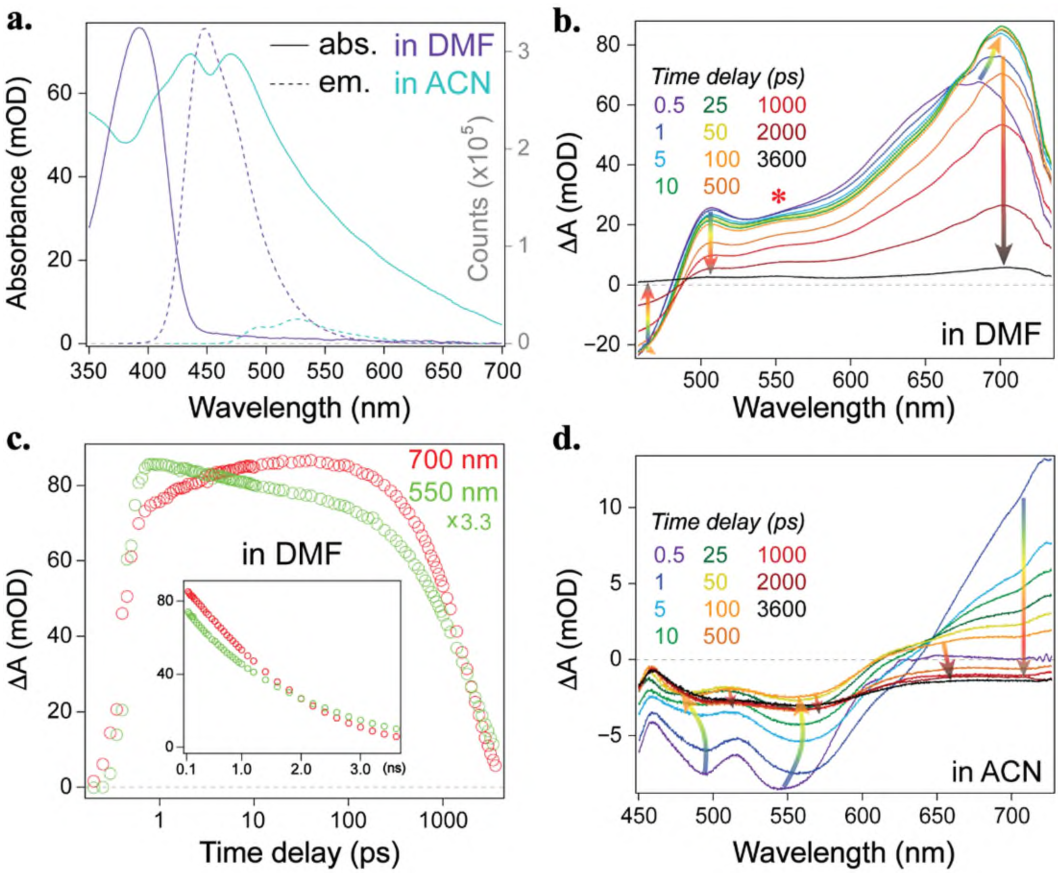

Supplementary Figure 9. Steady-state and transient electronic spectra of H_4TBAPy in solvated (DMF) and aggregated (acetonitrile, ACN) states. **a.** UV-visible absorption (solid) and emission (dashed) spectra of H_4TBAPy in DMF (purple) and ACN (cyan) solvents. Selective time points from fs-TA spectra of H_4TBAPy in **b.** DMF and **d.** ACN are color-coded, with the gradient arrows illustrating spectral evolution. The tilted downward arrows highlight the SE band rise on the hundreds of ps timescale. **c.** Probe-dependent dynamics at the 550 nm (green) and 700 nm (red) ESA bands of H_4TBAPy in DMF across the entire time window. The same data after 100 ps are plotted on a linear scale (in the inset) to highlight a longer lifetime of the 550 nm ESA band.

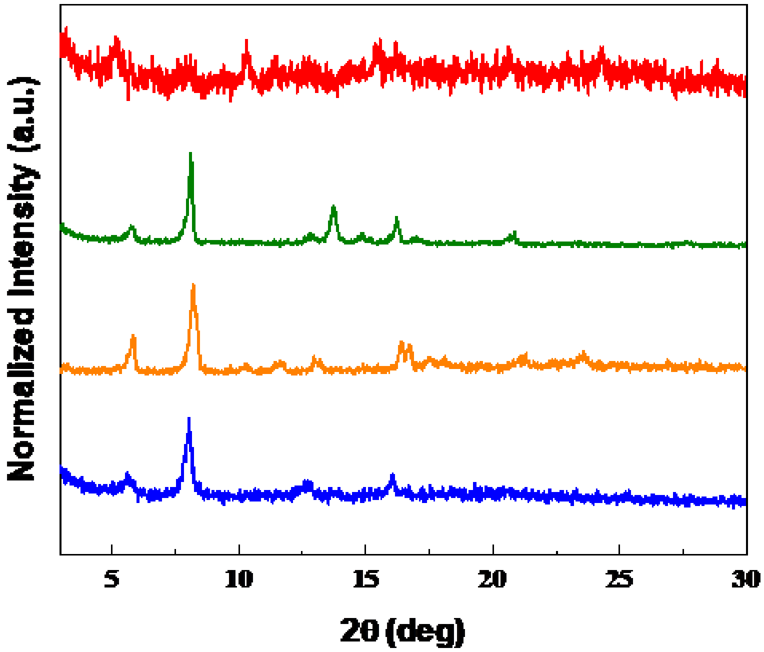

Supplementary Figure 10. PXRD patterns of MOFs post photocatalytic hydrogen evolution reaction. Sc-TBAPy (blue), Y-TBAPy (yellow), and Al-TBAPy (green) retained their crystalline structures after completing HER. Fe-TBAPy (red) lost its crystalline structure after performing HER.

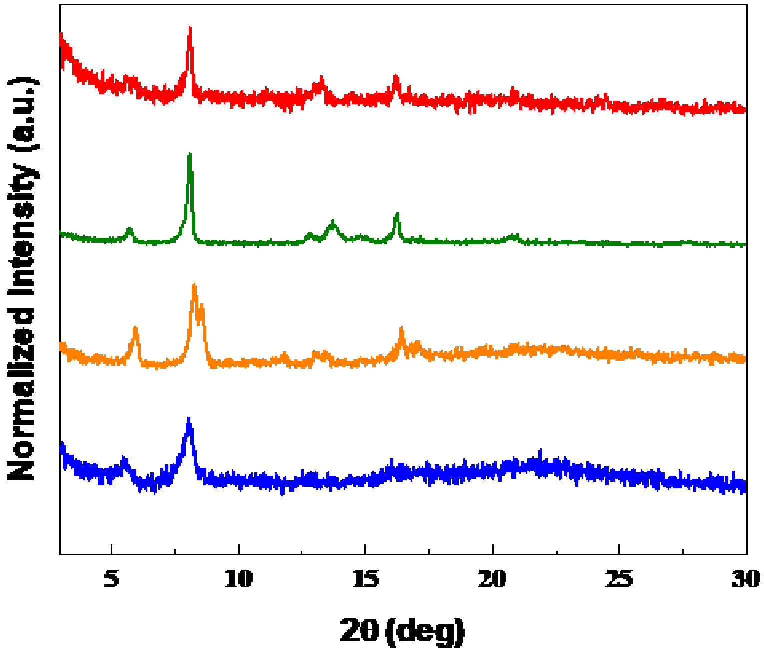

Supplementary Figure 11. PXRD patterns of MOFs post photocatalytic GP degradation reaction. All M^{3+}-TBAPy MOFs (Sc-TBAPy (blue), Y-TBAPy (yellow), Al-TBAPy (green), and Fe-TBAPy (red)) retained their crystalline structures after the reaction.

a

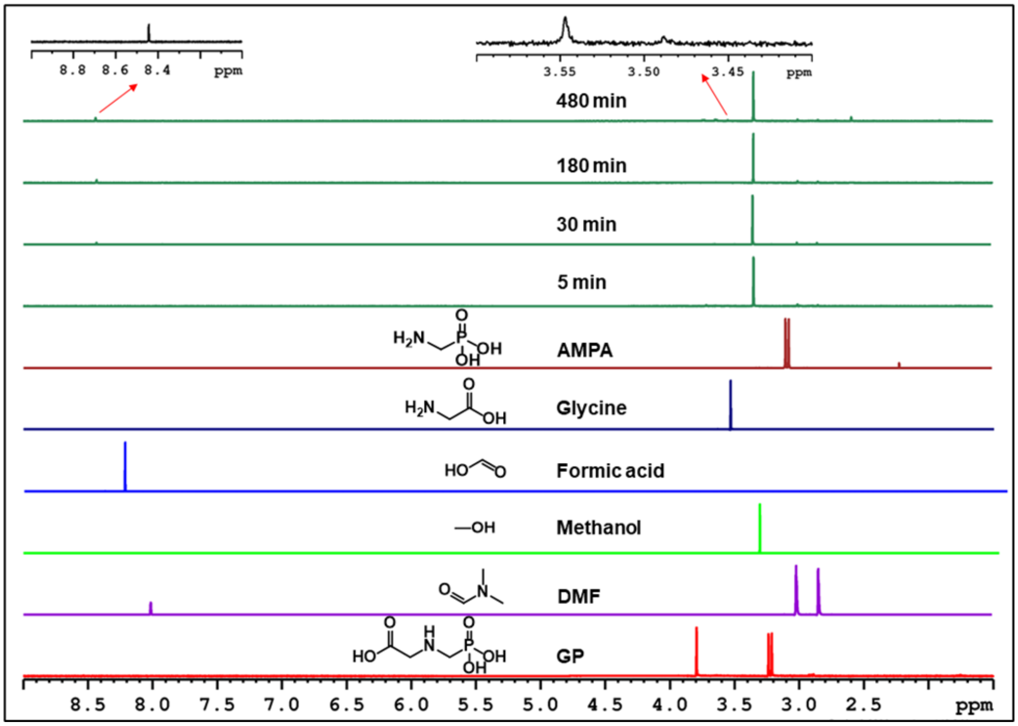

480 min
180 min
30 min
5 min
AMPA
Glycine
Formic acid
Methanol
DMF
GP
ppm

b

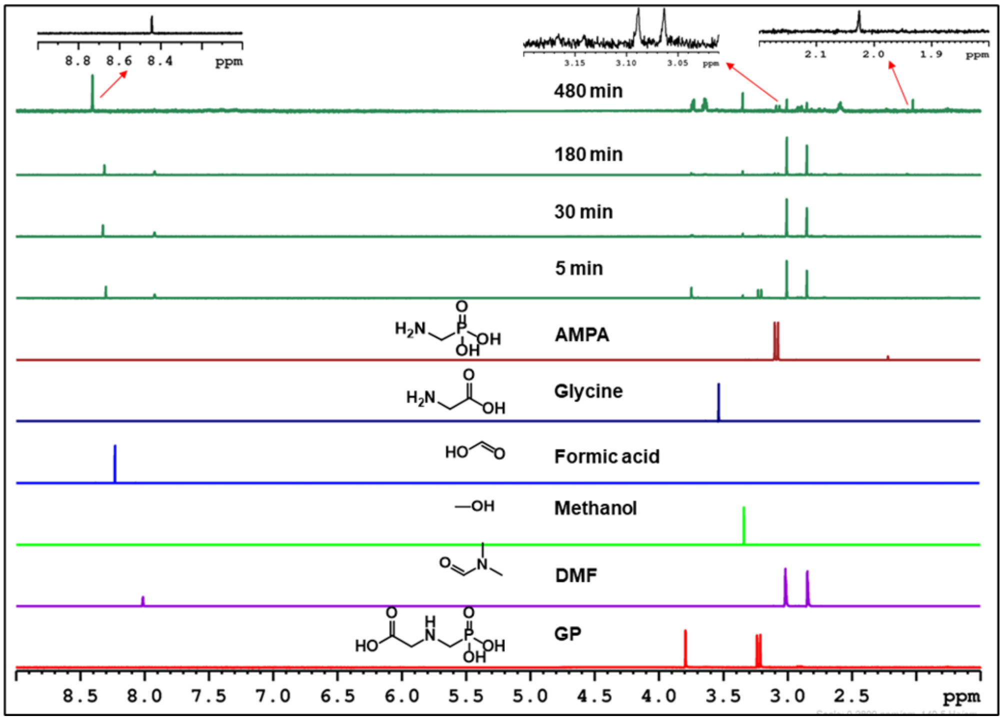

480 min
180 min
30 min
5 min
AMPA
Glycine
Formic acid
Methanol
DMF
GP
ppm

c

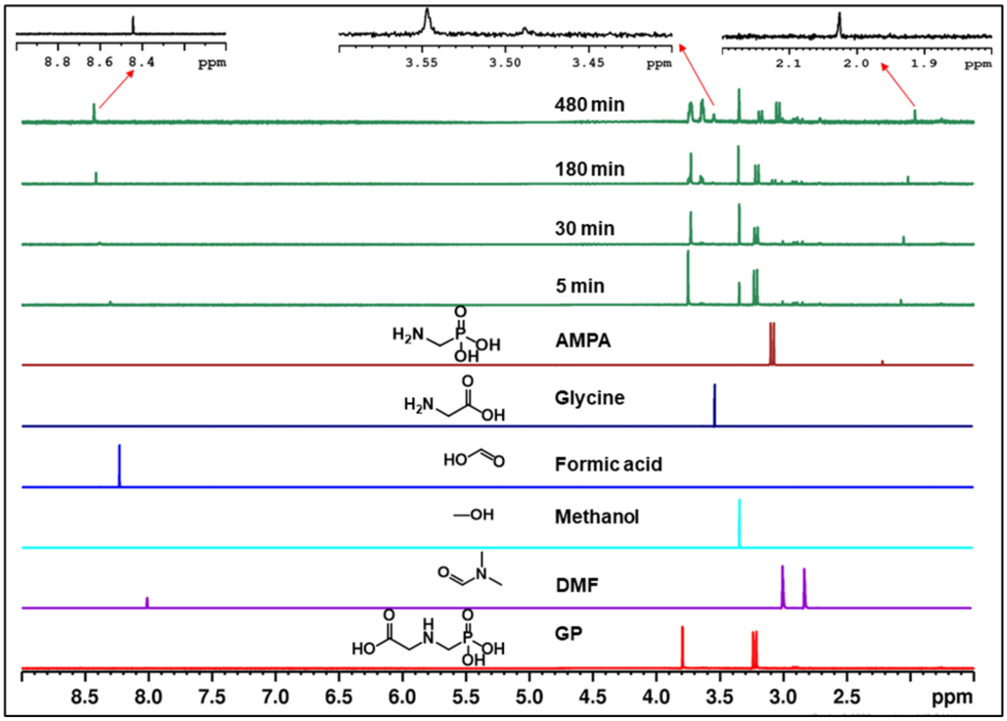

480 min
180 min
30 min
5 min
AMPA
Glycine
Formic acid
Methanol
DMF
GP
8.5
8.0
7.5
7.0
6.5
6.0
5.5
5.0
4.5
4.0
3.5
3.0
2.5
ppm

d

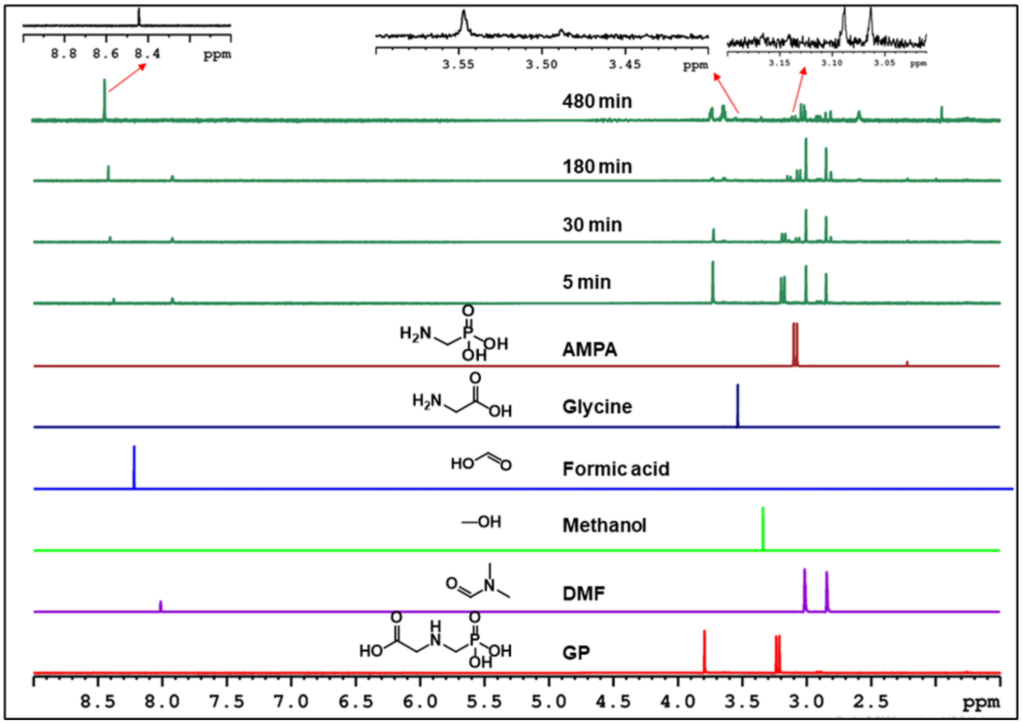

480 min
180 min
30 min
5 min
AMPA
Glycine
Formic acid
Methanol
DMF
GP
8.5
8.0
7.5
7.0
6.5
6.0
5.5
5.0
4.5
4.0
3.5
3.0
2.5
ppm

Supplementary Figure 12. ^{1}H NMR spectra for standards (GP, glycine, methanol, and DM), and products of GP degradation half reaction after 8 h of irradiation. In the presence of **a.** Sc-TBAPy, only formic acid [δ 8.36 ppm (singlet)] and glycine [δ 3.54 ppm (singlet)] were observed. In contrast, the spectra show the emergence of formic acid [δ 8.36 ppm (singlet)], glycine [δ 3.54 ppm (singlet)], AMPA [δ 3.09 ppm and 3.07 ppm (doublet)] with GP still present in the solution after 8 hr when **b.** Al-TBAPy **c.** Fe-TBAPy and **d.** Y-TBAPy were used as the photocatalyst. The peaks at [δ 7.92 ppm (singlet)] and [δ 3.01 ppm and 2.85 ppm (doublet)] were assigned to DMF, and the peak at [δ 3.35 ppm (singlet)] was assigned to methanol. All these peaks were attributed to have emerged from the MOFs as solvents used in washing the MOFs. A small peak at [δ 2.06 ppm (singlet)] was also observed and assigned to acetic acid. Slight variations of the chemical shifts (i.e., peak positions) of glycine, AMPA and formic acid were attributed to the changing pH over the course of the irradiation due to the production of phosphoric acid (H_3PO_4).

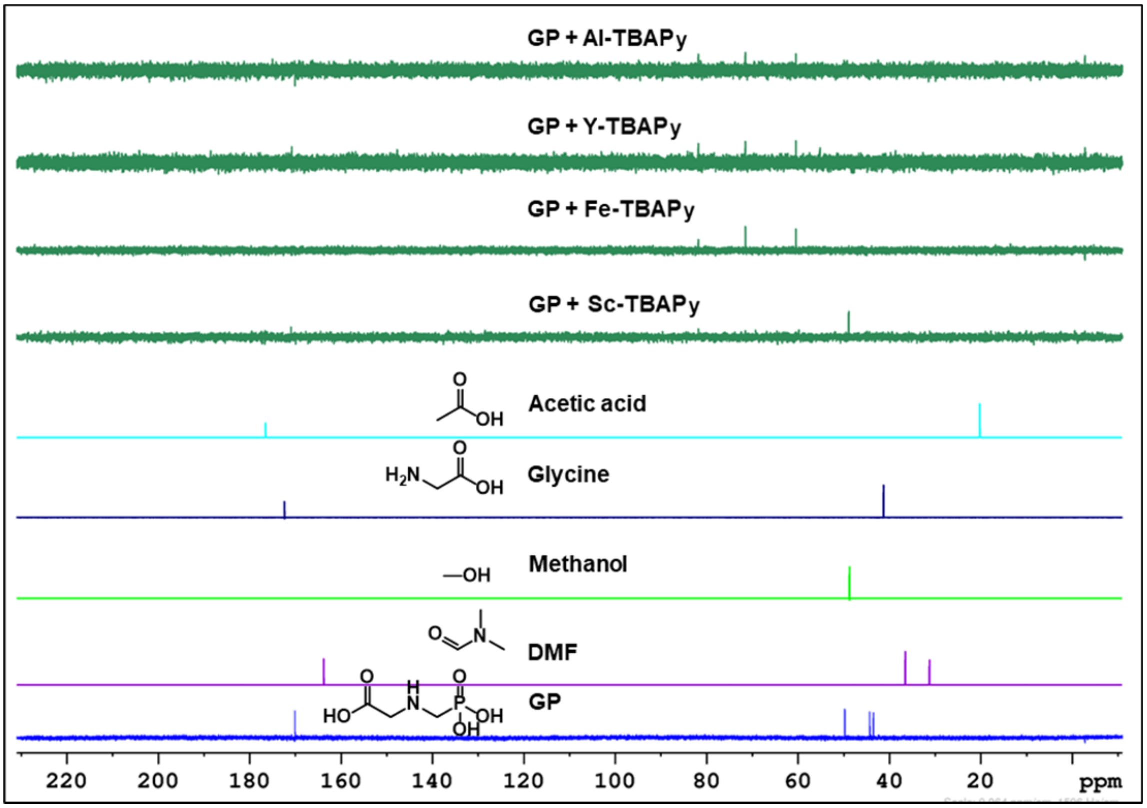

Supplementary Figure 13. ^{13}C NMR of standards (GP, DMF, methanol, glycine, and acetic acid) and products of GP degradation after 8 hr. No sign of GP was detected in the NMR spectra for all four MOFs. It is speculated that the concentration of the possible products from GP degradation may have been below the detection limit of the ^{13}C NMR. The peak at [δ 48.86 ppm (singlet)] was assigned to methanol and was attributed to have emerged from the MOF.

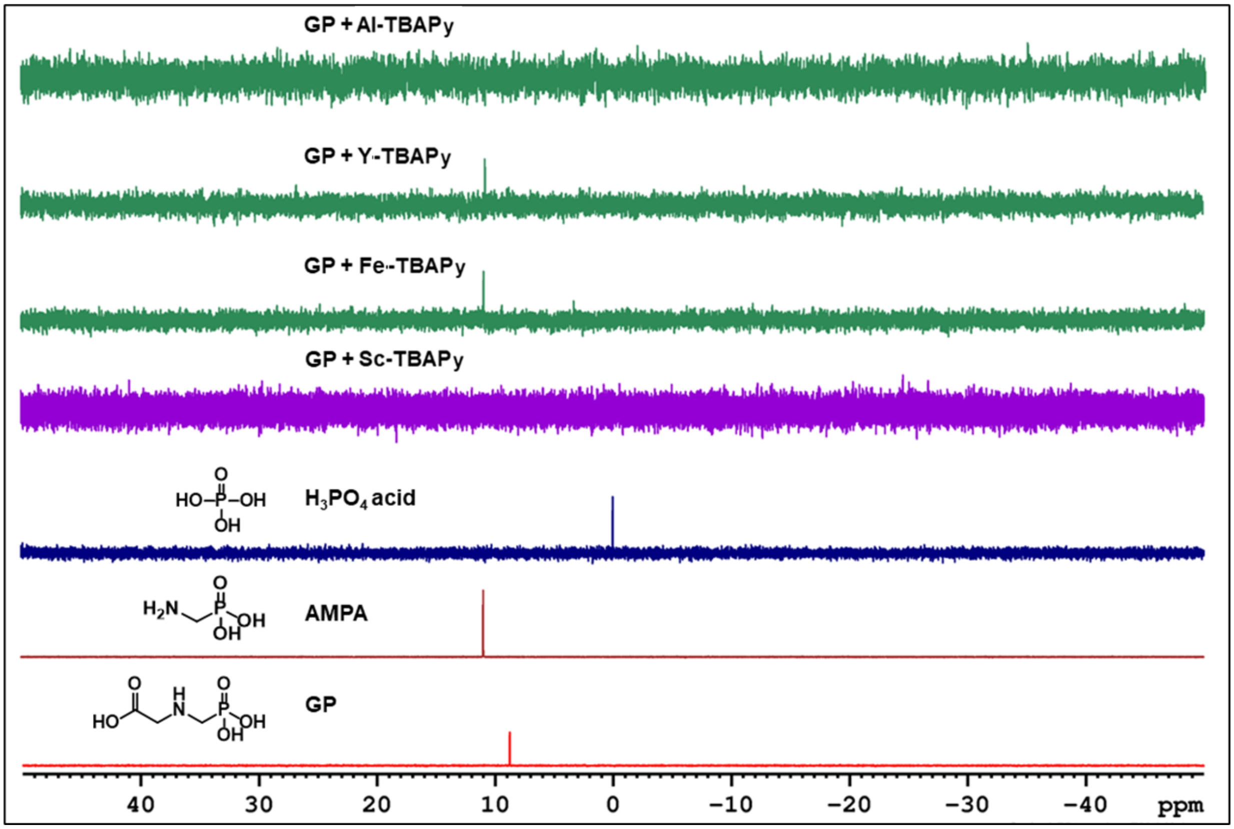

Supplementary Figure 14. ^{31}P NMR spectra of the standards (GP, AMPA, and phosphoric acid), and products of GP degradation after 8 hr. No trace of GP or phosphoric acid was detected in the NMR spectra for all four MOFs. Only AMPA with a peak at [δ 10.94 ppm (singlet)] was detected for Fe-TBAPy and Y-TBAPy.

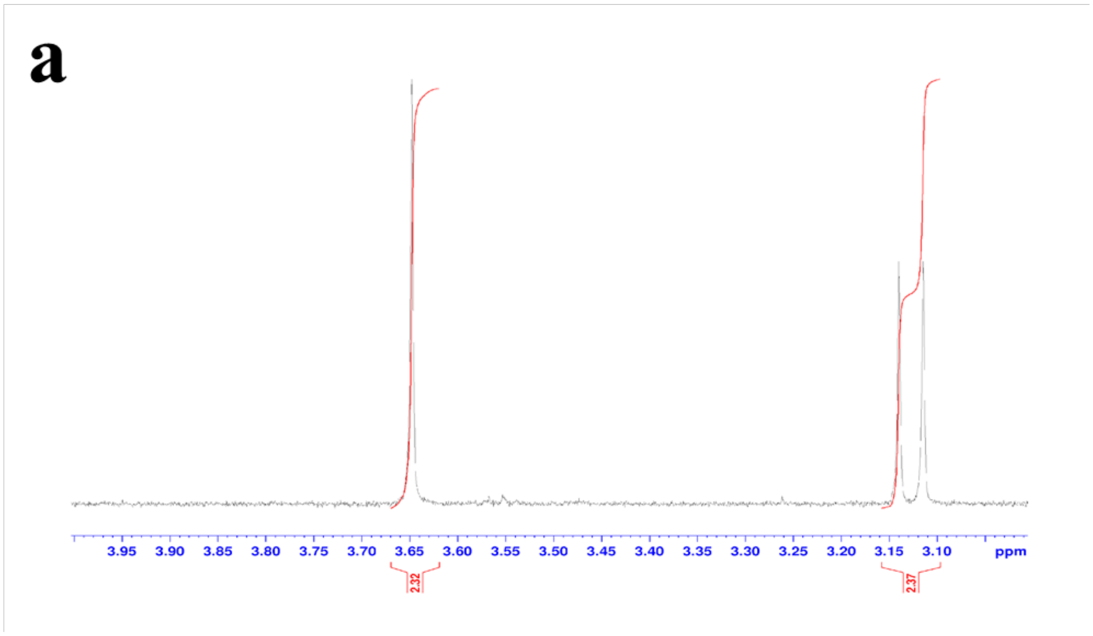
a
3.95 3.90 3.85 3.80 3.75 3.70 3.65 3.60 3.55 3.50 3.45 3.40 3.35 3.30 3.25 3.20 3.15 3.10 ppm
2.32
2.37

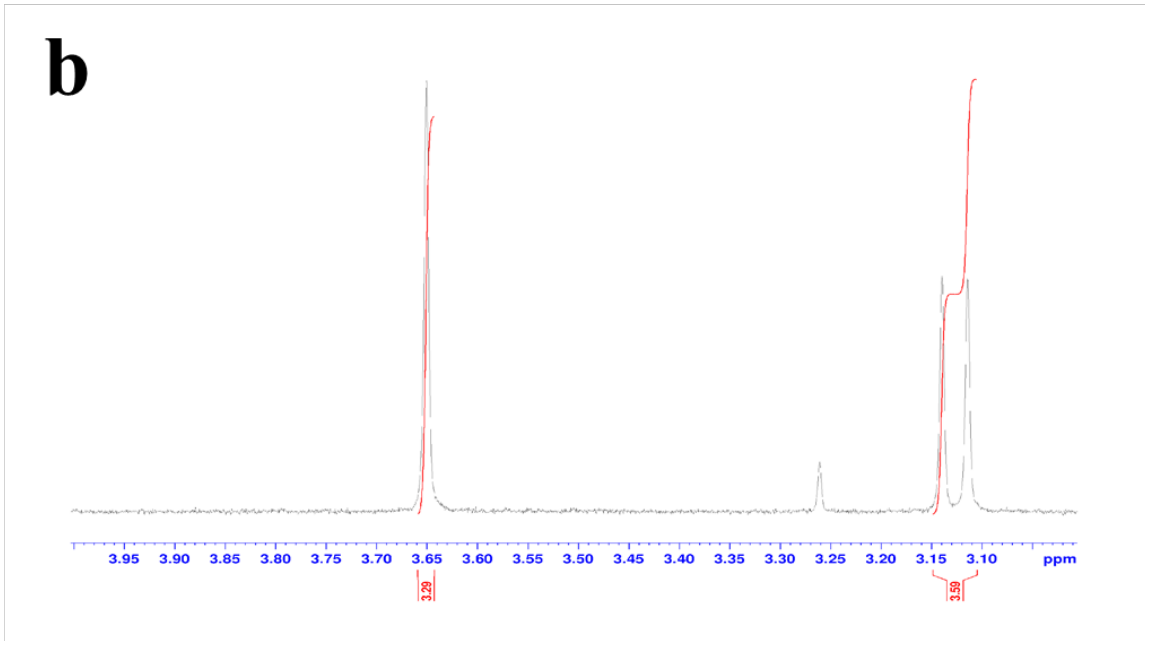
b
3.95 3.90 3.85 3.80 3.75 3.70 3.65 3.60 3.55 3.50 3.45 3.40 3.35 3.30 3.25 3.20 3.15 3.10 ppm
3.29
3.59

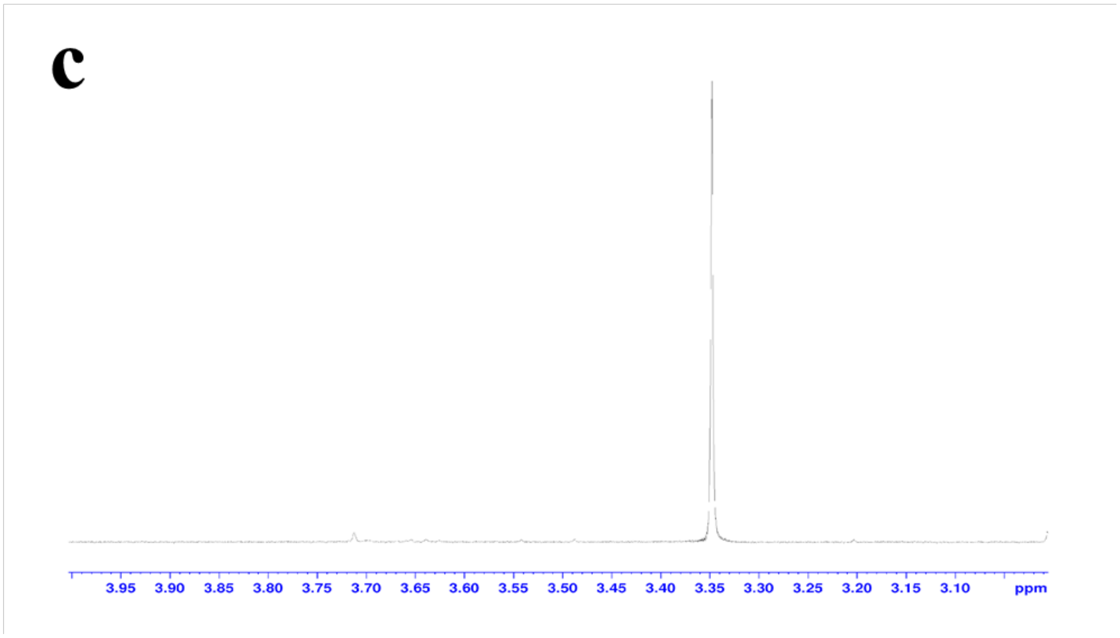

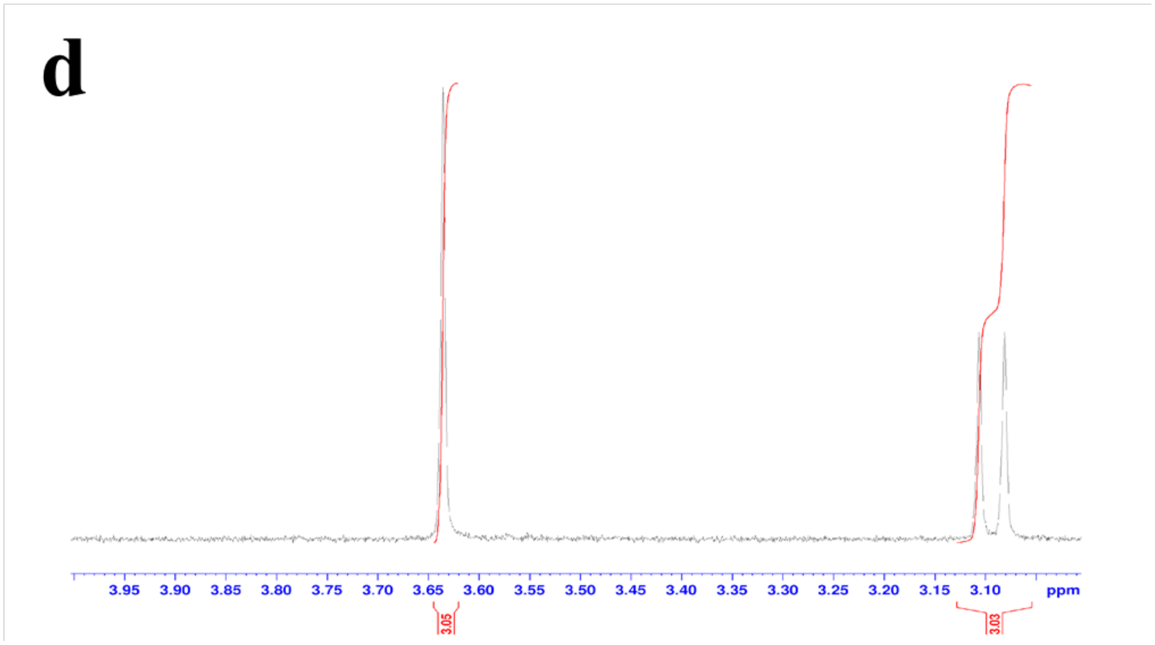

Supplementary Figure 15. ^{1}H NMR spectra of GP solution (initial concentration 1.5 mM) after exposure to **a.** Al-TBAPy, **b.** Fe-TBAPy, **c.** Sc-TBAPy, and **d.** Y-TBAPy for 5 min in the absence of light. Percent uptakes were calculated by comparing integration values against those of a 1.5 mM GP standard solution.

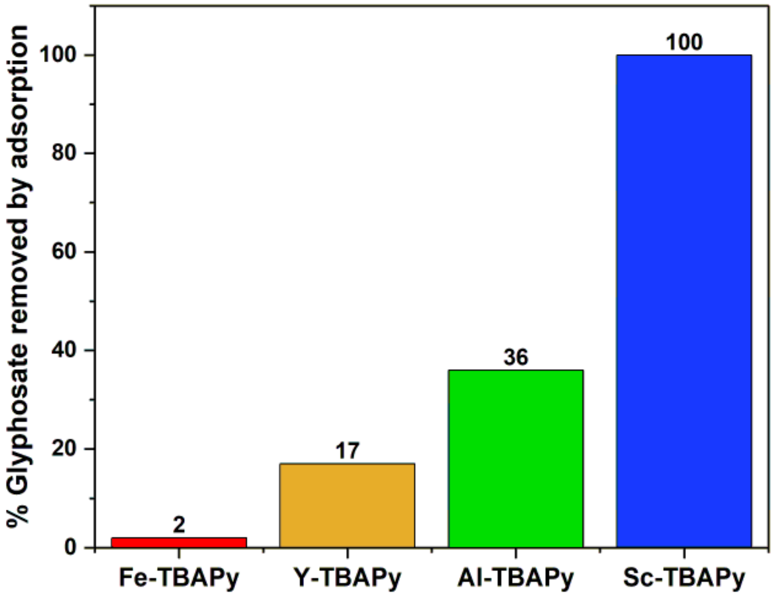

Supplementary Figure 16. Percent GP uptakes by the four M^{3+}-TBAPy MOFs after 5 minutes.

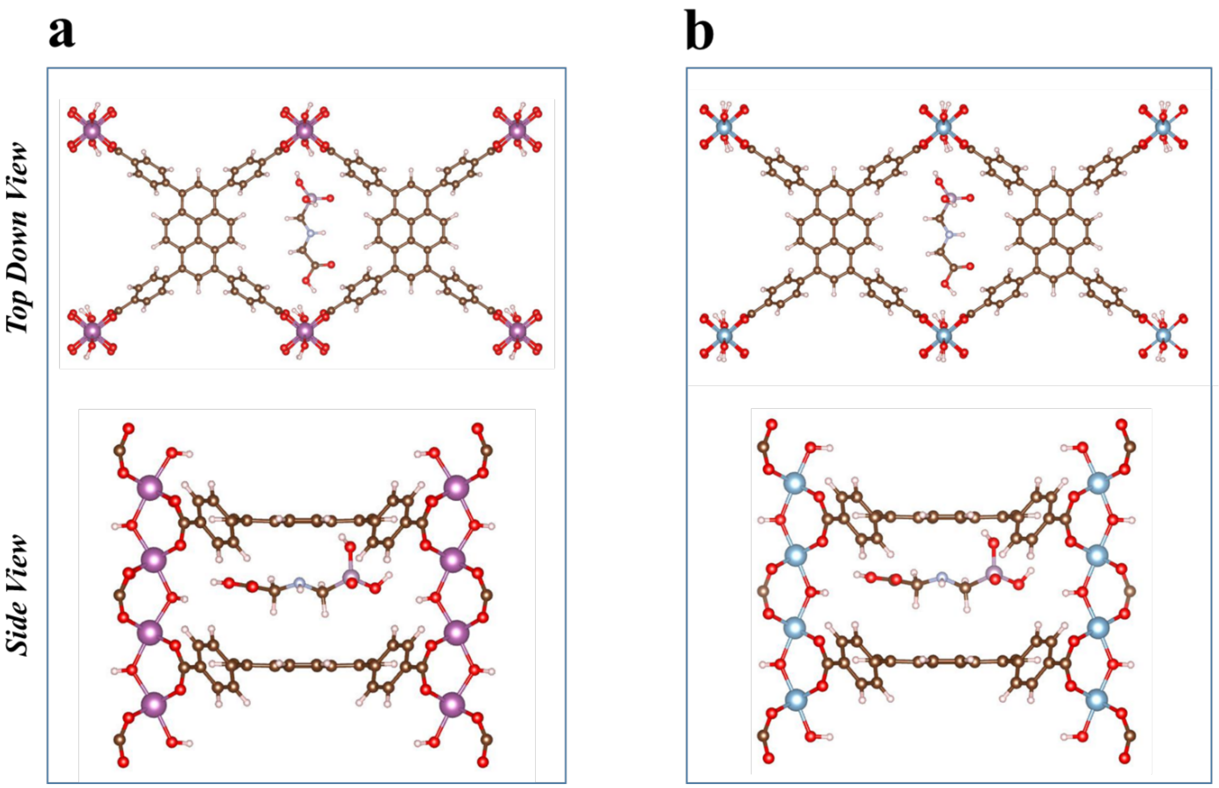

Supplementary Figure 17. Computationally derived orientations of GP within pore B of **a.** Sc-TBAPy and **b.** Al-TBAPy. DFT calculations employed a mixed Gaussian and planewave basis sets. Atom colors: magenta, Sc^{3+}; cyan, Al^{3+}; brown, C; red, O; white, H.

Supplementary Table 3. Binding energies for Sc-TBAPy and Al-TBAPy for GP with three accessible pores (A, B, and C, defined in Figure 1 in main text).

MOF	Pore	ΔE (kJ/mol)
Sc-TBAPy	A	– 93.6
	B	– 98.6
	C	– 94.8
Al-TBAPy	A	– 77.2
	B	– 78.0
	C	– 75.9

a

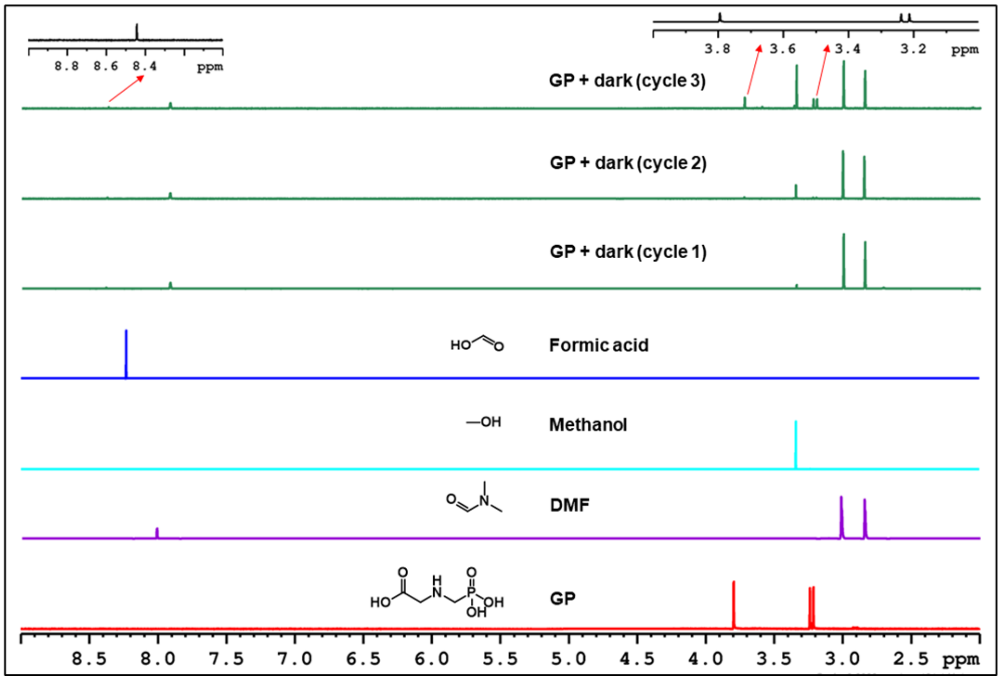

GP + dark (cycle 3)
GP + dark (cycle 2)
GP + dark (cycle 1)
Formic acid
Methanol
DMF
GP
8.5 8.0 7.5 7.0 6.5 6.0 5.5 5.0 4.5 4.0 3.5 3.0 2.5 ppm
8.8 8.6 8.4 ppm
3.8 3.6 3.4 3.2 ppm

b

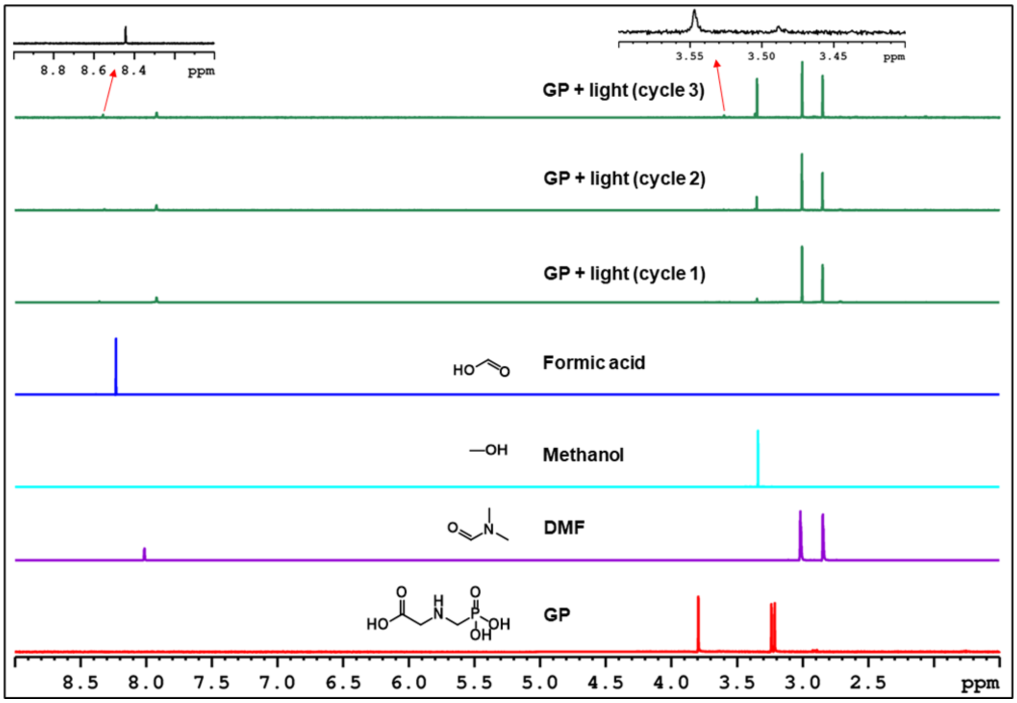

GP + light (cycle 3)
GP + light (cycle 2)
GP + light (cycle 1)
Formic acid
Methanol
DMF
GP
8.5 8.0 7.5 7.0 6.5 6.0 5.5 5.0 4.5 4.0 3.5 3.0 2.5 ppm
8.8 8.6 8.4 ppm
3.55 3.50 3.45 ppm

c

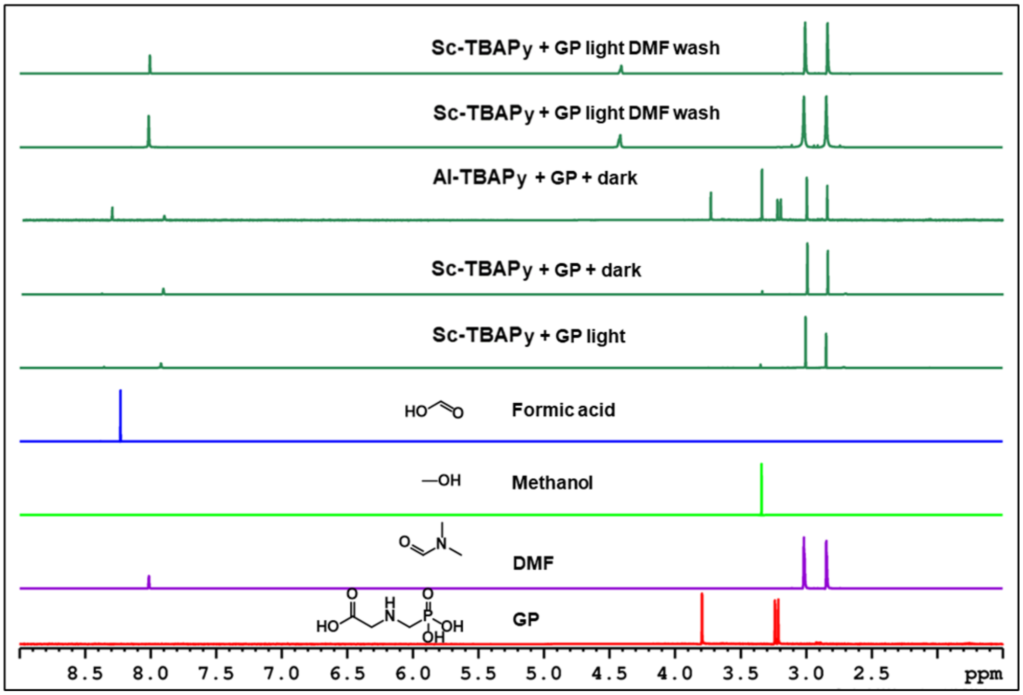

Supplementary Figure 18. ¹H NMR spectra for standards (GP, glycine, methanol, and DMF), and products of GP degradation half-reaction after 3 consecutive cycles with Sc-TBAPy and Al-TBAPy. **a.** Using Sc-TBAPy, under dark conditions, only a small amount of formic acid [δ 8.36 ppm (singlet)] was observed, with no GP present. GP remained present in the solution after cycles 2 and 3, indicating the saturation of MOF pores in the solution after cycle 1. **b.** Using Sc-TBAPy, under light, only formic acid [δ 8.36 ppm (singlet)] and glycine [δ 3.54 ppm (singlet)] were observed for all 3 cycles, with no evidence of GP in the solution. The peaks at [δ 7.92 ppm (singlet)] and [δ 3.01 ppm and 2.85 ppm (doublet)] were assigned to DMF, and the peak at [δ 3.35 ppm (singlet)] was assigned to methanol. Both peaks were believed to have emerged from the MOFs as solvents used in washing the MOFs. **c.** Sc-TBAPy washed with DMF solution after oxidation reaction under light and dark conditions for 5 minutes. In the case of Al-TBAPy, a large amount of GP remained to be unreacted under similar dark reaction conditions as Sc-TBAPy.

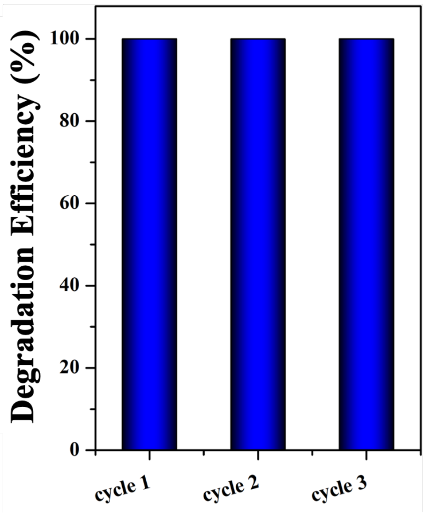

Supplementary Figure 19. Recyclability tests showing the degradation of GP under UV-light for over three consecutive cycles using Sc-TBAPy.

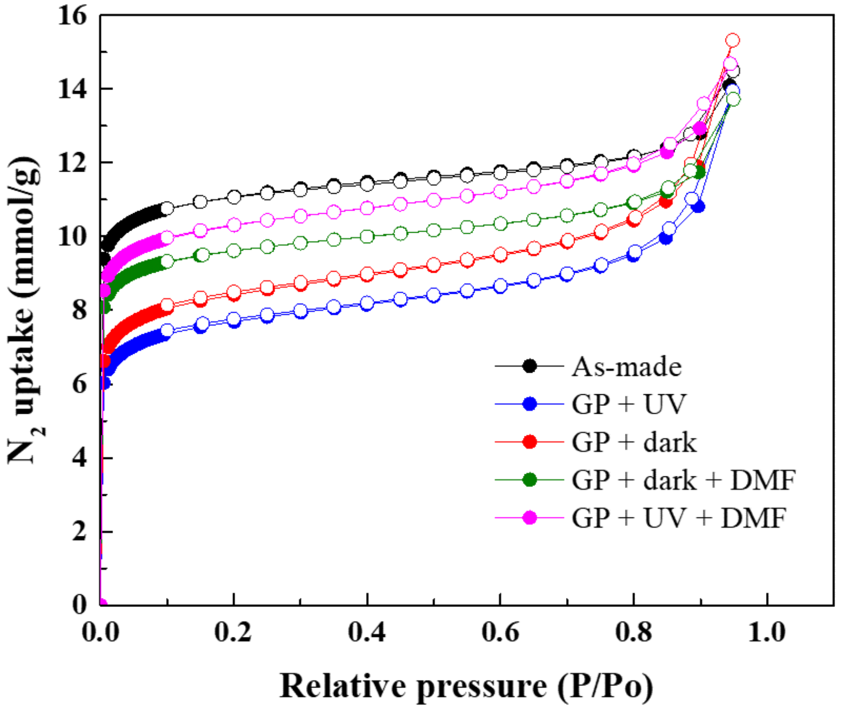

Supplementary Figure 20. Nitrogen adsorption isotherms for the as-made Sc-TBAPy and after oxidation reaction with GP for 5 minutes under dark and light conditions and after washing with DMF solution.

Supplementary Table 4. BET surface area of the as-made Sc-TBAPy and Sc-TBAPy after oxidation reaction with GP for 5 minutes under dark and light conditions and after washing with DMF solution.

Sample	Surface area ($m^2\ g^{-1}$)
As-made	793
GP in UV light	654
GP in dark	714
GP in dark, DMF washed	832
GP in UV light, DMF washed	884

Our results indicated the presence of 1.6 ppm of Sc^{3+} in the solution, corresponding to approximately 0.42% degradation of Sc-TBAPy. This suggests minimal leaching of Sc^{3+} from Sc-TBAPy during the degradation process. This slight Sc^{3+} leaching might potentially generate defects within the Sc-TBAPy structure, leading to an increase in the BET surface area of Sc-TBAPy.

a

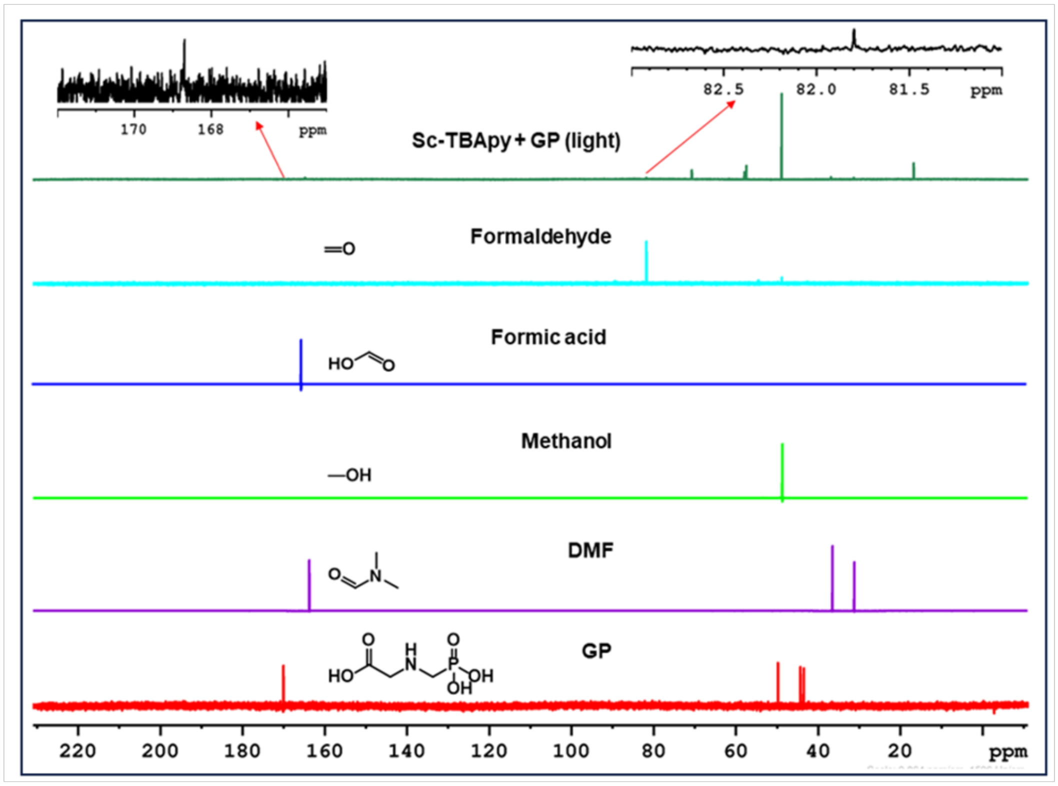

b

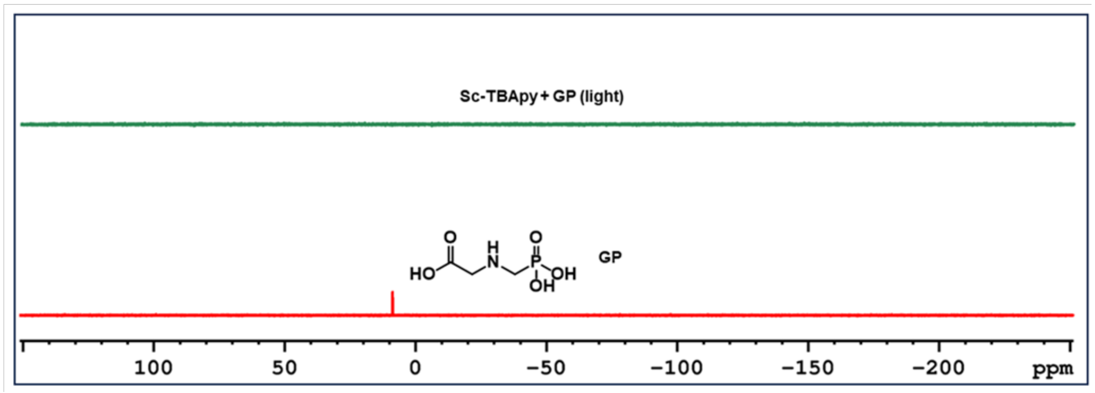

Supplementary Figure 21. NMR spectra of the standards (GP, DMF, methanol, formaldehyde, and formic acid), and products of GP degradation half-reaction after the third cycle with Sc-TBAPy. **a.** ^{13}C NMR shows only formic acid [δ 168.7 ppm (singlet)], with no evidence of GP in the solution. The peaks at [δ 164.8 ppm (singlet)] and [δ 36.8 ppm and 31.3 ppm (doublet)] were assigned to DMF, and the peak at [δ 48.8 ppm (singlet)] was assigned to methanol. Both DMF and methanol peaks were believed to have emerged from solvents used in washing the MOF. Note: the

peak at [δ 81.7 ppm (singlet)] was assigned to formaldehyde, which is a reduced product of formic acid. Glycine was undetected in the ^{13}C NMR spectra due to its low concentration in solution. Other unassigned peaks were believed to have emerged from impurities. **b.** ^{31}P NMR shows no evidence of GP or any product of GP degradation such as phosphoric acid in solution. It was considered that the concentrations of possible products may have been low, hence undetected in the ^{31}P NMR.

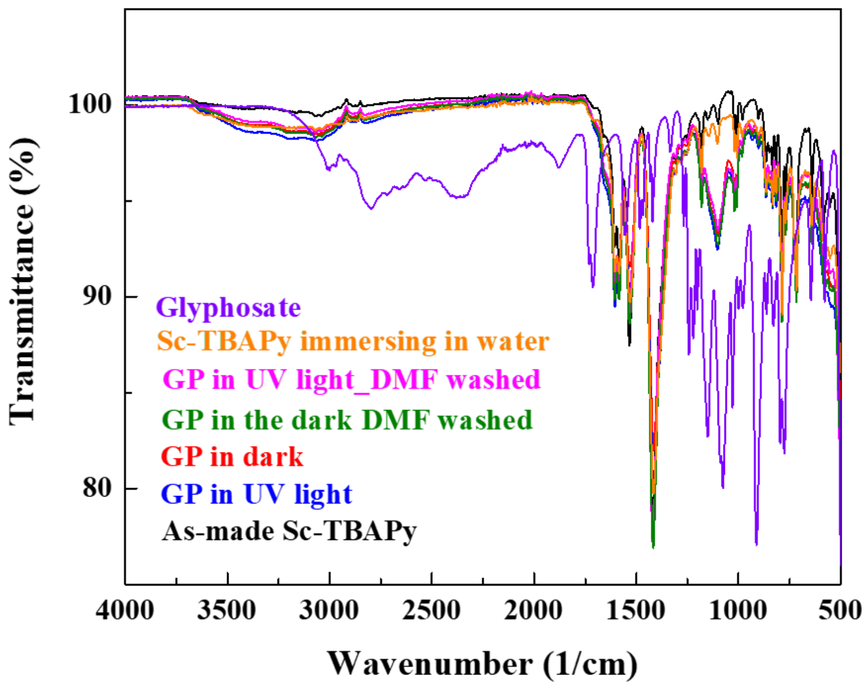

Supplementary Figure 22. FTIR spectra for the as-made Sc-TBAPy and Sc-TBAPy after oxidation reaction of GP for 5 minutes under dark and light conditions and after washing with DMF solution. The FTIR spectra show that the post reaction Sc-TBAPy (yellow) retains the structure of as-made MOF (black) before the oxidation reactions.

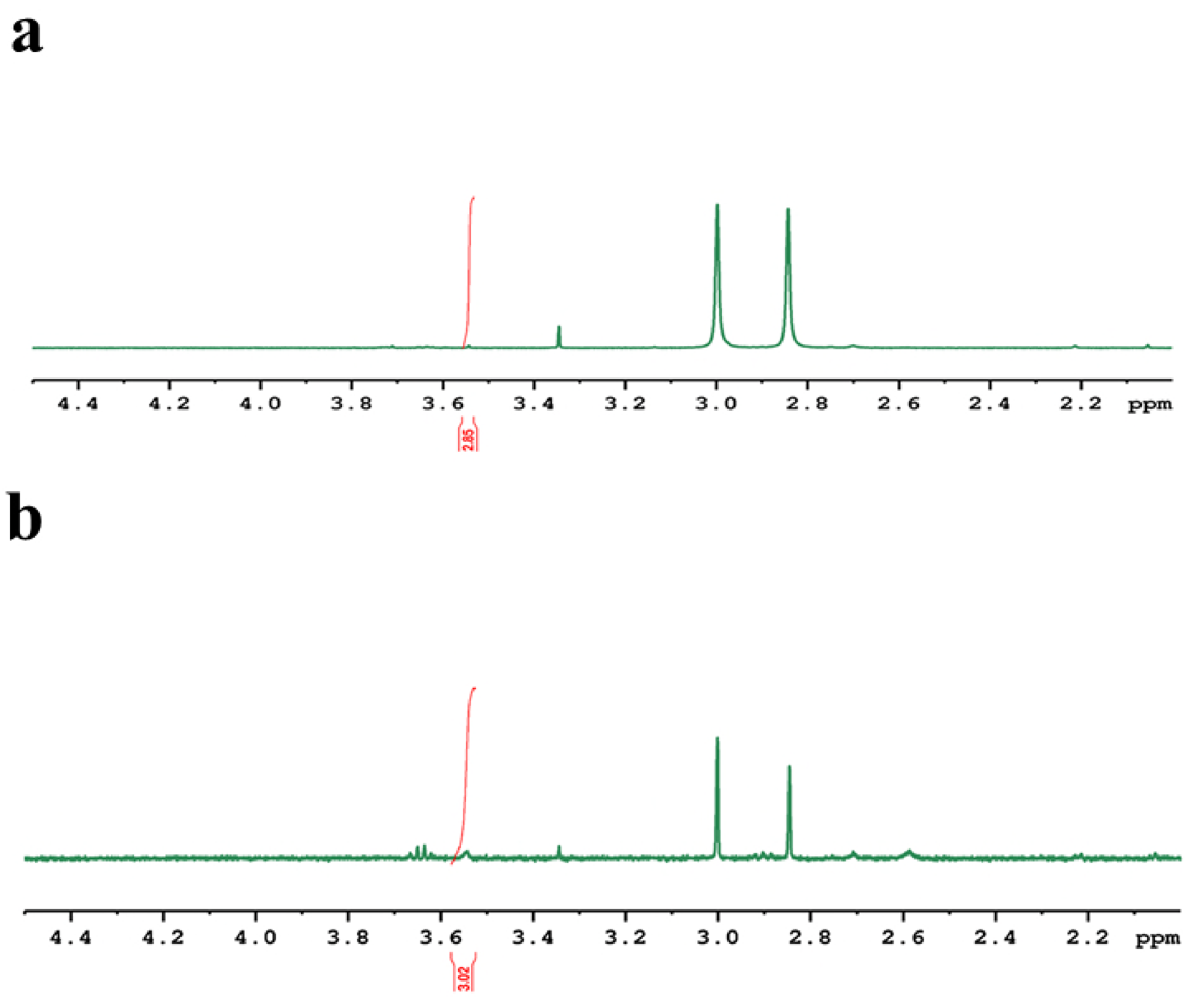

Supplementary Figure 23. ^{1}H NMR spectra of **a.** pH 10.7 GP solution and **b.** GP solution in river water after exposure to Sc-TBAPy. Only formic acid [δ 8.36 ppm (singlet)] and glycine [δ 3.54 ppm (singlet)] were observed with no evidence of GP in the solution.

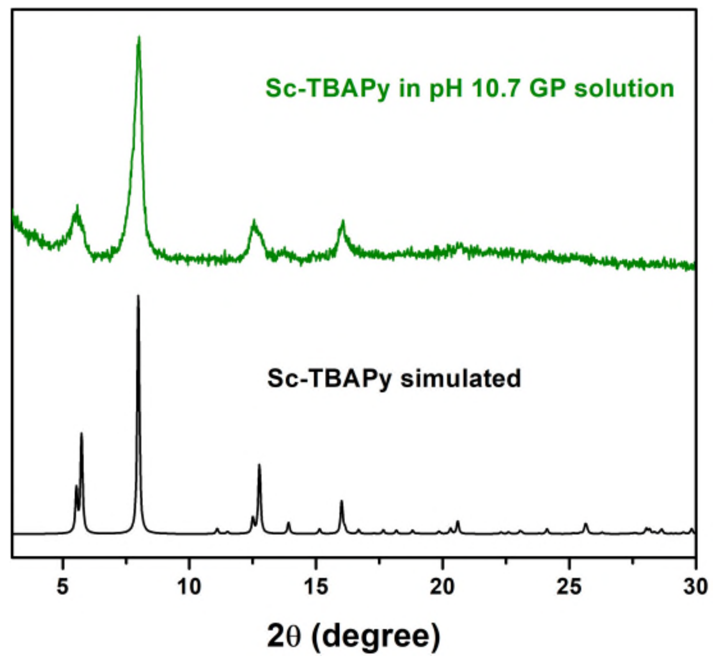

Supplementary Figure 24. PXRD patterns collected on Sc-TBAPy after photocatalytic pH 10.7 GP degradation indicate that the MOF maintained its crystalline structure after the reaction.

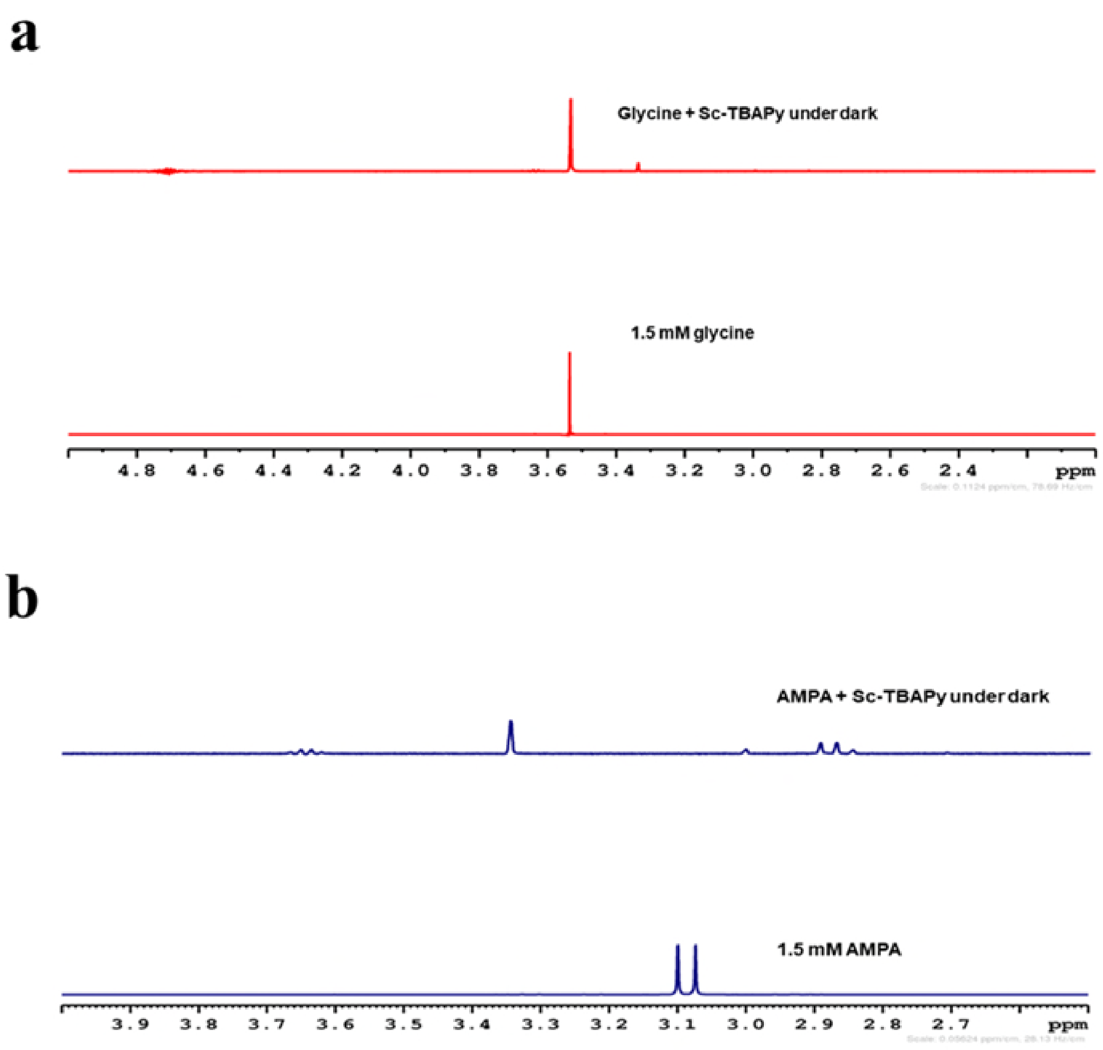

Supplementary Figure 25. **^{1}H NMR spectra** of the **a.** glycine solution and **b.** AMPA solution, before and after exposure to Sc-TBAPy under dark conditions for 5 min, reveal that glycine remained in the solution, whereas AMPA completely disappeared. This indicates the capacity of the MOF to adsorb toxic metabolites of GP.

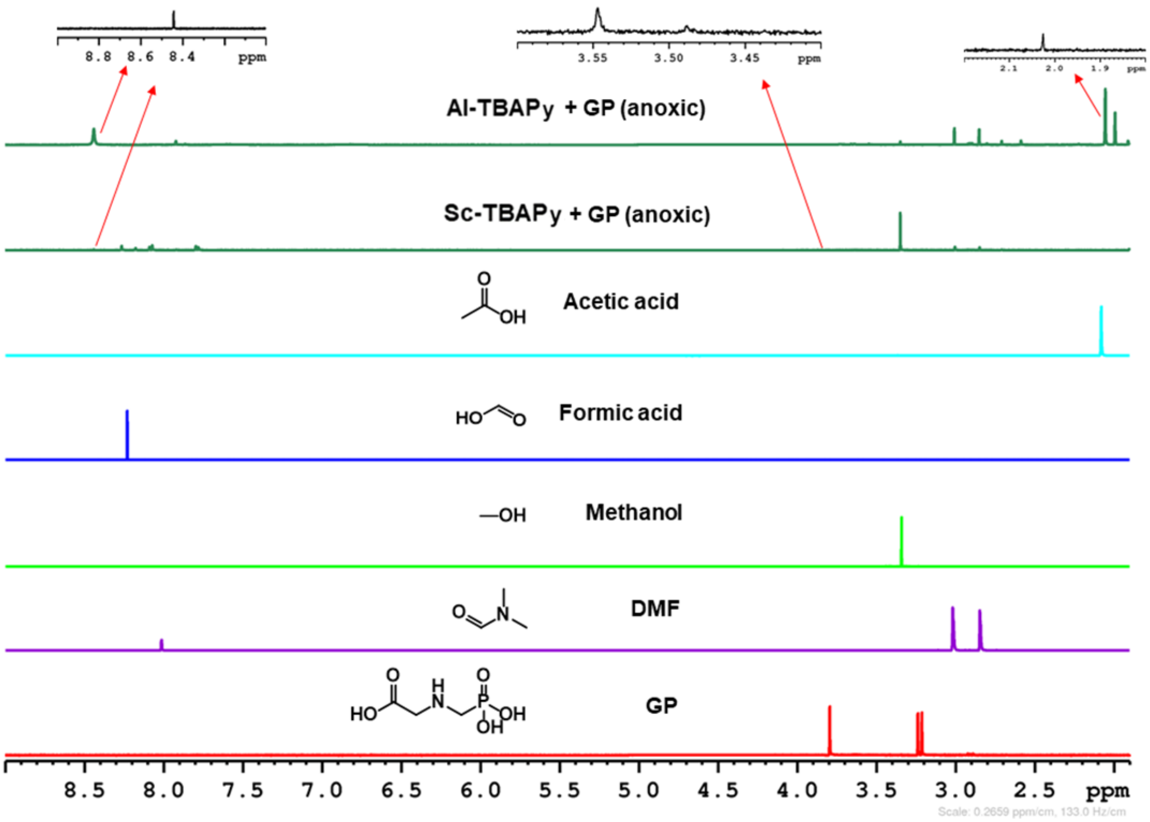

Supplementary Figure 26. ^{1}H NMR spectra for standards (GP, DMF, methanol, formic acid, and acetic acid), and products of GP degradation under anoxic condition using Sc-TBAPy and Al-TBAPy. Under anoxic conditions, only formic acid [δ 8.33 ppm (singlet)] and glycine [δ 3.54 ppm (singlet)] were observed, when Sc-TBAPy was used as the catalyst. For Al-TBAPy, only formic acid and acetic acid [δ 2.06 ppm (singlet)] were present. Both MOFs showed no trace of GP under anoxic conditions.

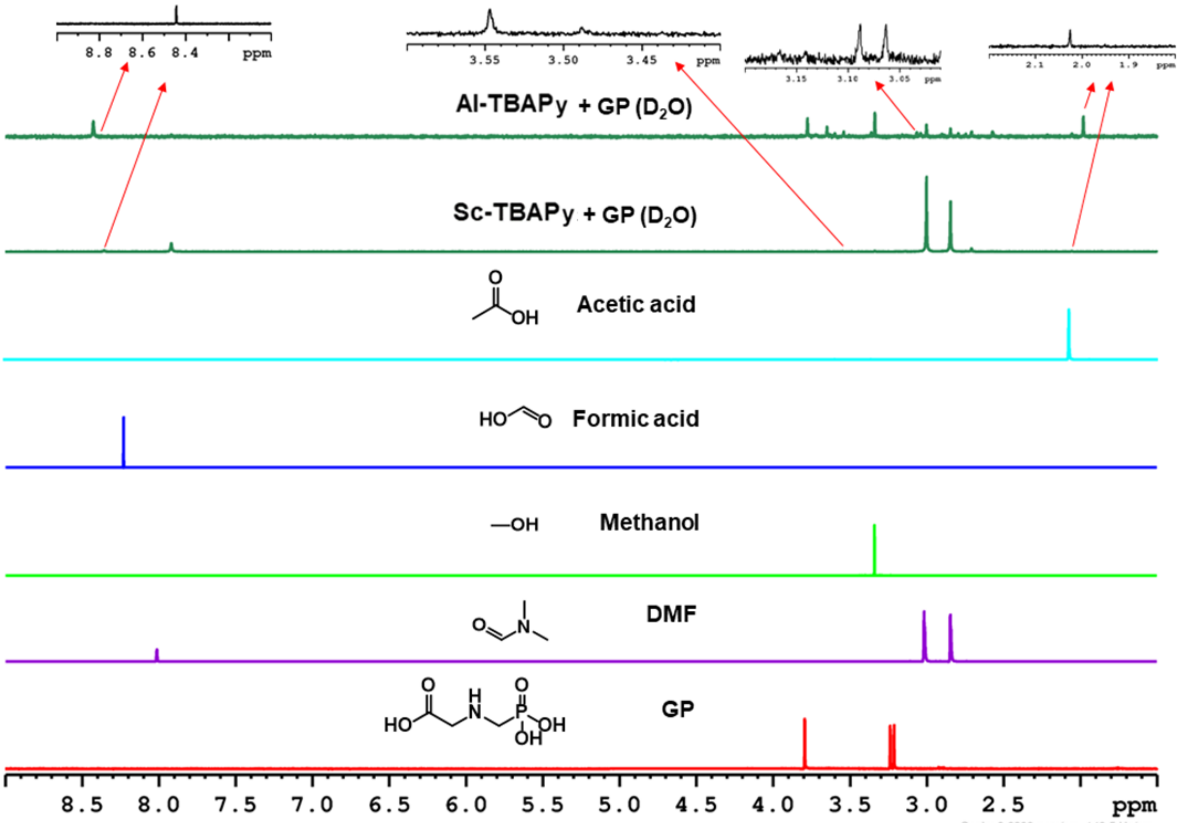

Supplementary Figure 27. ^{1}**H NMR spectra for standards (GP, DMF, methanol, formic acid, and acetic acid), and products of GP degradation in D_2O** using Sc-TBAPy and Al-TBAPy. In the presence of D_2O as the reaction solvent, only formic acid, glycine, and traces of acetic acid were detected using Sc-TBAPy. For Al-TBAPy, formic acid and acetic acid were also detected, with traces of AMPA [δ 3.09 ppm and 3.07 ppm (doublet)] also observed in the solution. Both MOFs showed no trace of GP under in the D_2O reaction. The peaks at [δ 7.92 ppm (singlet)] and [δ 3.01 ppm and 2.85 ppm (doublet)] were assigned to DMF, and the peak at [δ 3.35 ppm (singlet)] was assigned to methanol. Both peaks were believed to have emerged from the MOFs (solvents used in washing the MOFs).

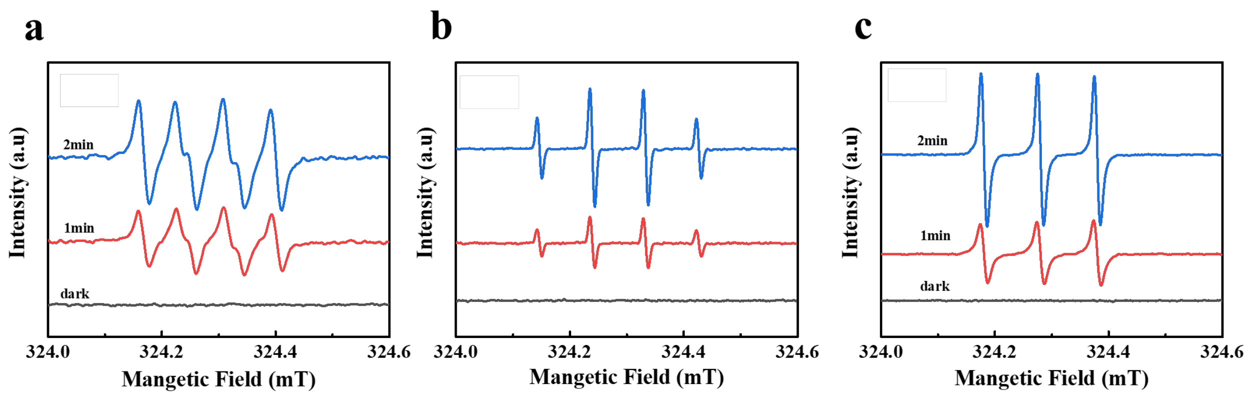

Supplementary Figure 28. Electron spin resonance (ESR) spectroscopy of **a.** $O_2^{\bullet-}$, **b.** •OH, and **c.** $^1O_2$ ROS formation with Sc-TBAPy. Without light irradiation, no ROS signal is detected (dark). With light irradiation, the intensity of ROS signals in the $O_2^{\bullet-}$, •OH, and $^1O_2$ spectra increased with longer irradiation time from 1 min (red) to 2 min (blue).

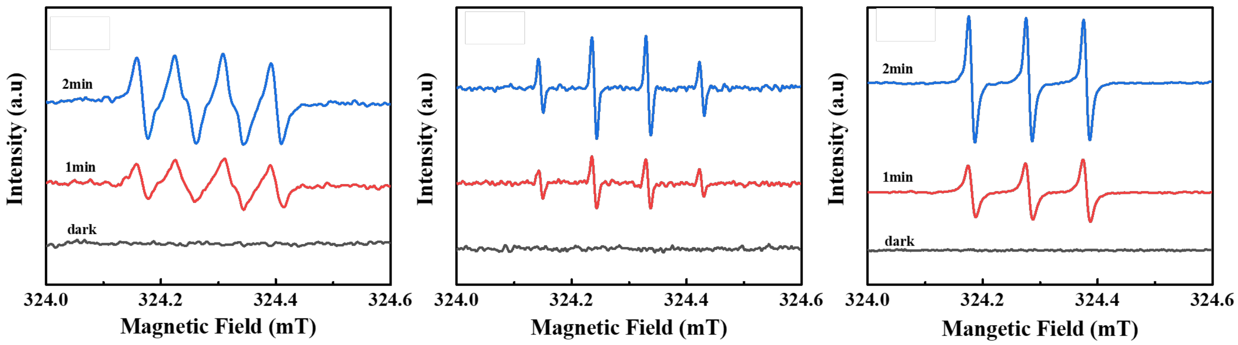

Supplementary Figure 29. ESR spectroscopy of **a.** $O_2^{\bullet-}$, **b.** •OH, and **c.** $^1O_2$ ROS formation with Al-TBAPy. Without light irradiation, no ROS signal is detected (dark). With light irradiation, the intensity of ROS signals in the $O_2^{\bullet-}$, •OH, and $^1O_2$ spectra increased with longer irradiation time from 1 min (red) to 2 min (blue).

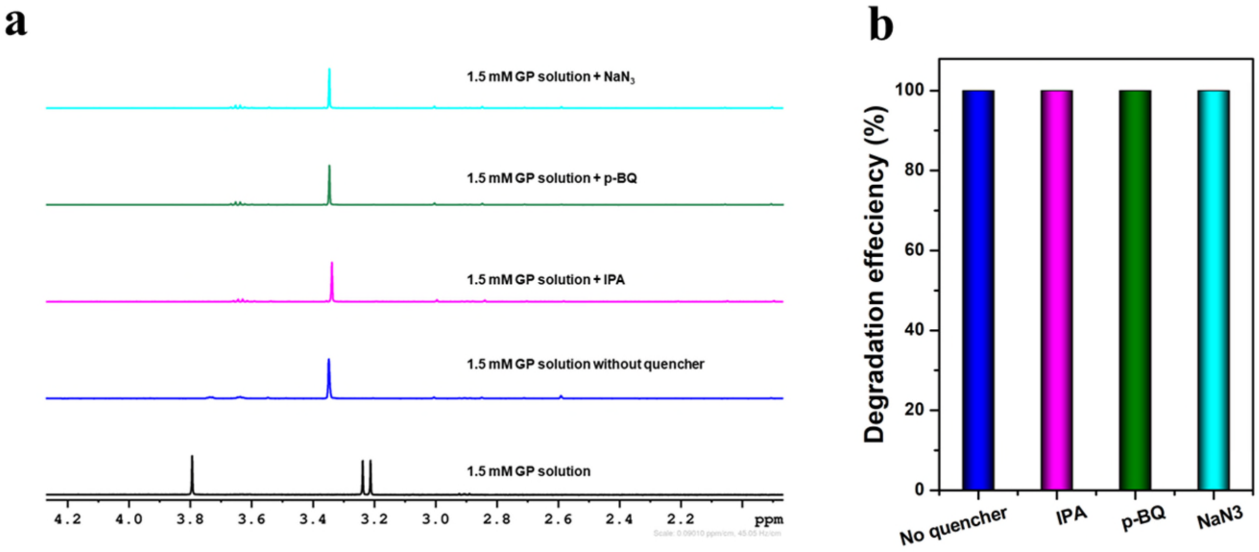

Supplementary Figure 30. a. ^{1}H NMR spectra for pure 1,5 mM GP solution after degradation reaction in the presence of ROS scavengers using Sc-TBAPy. The spectra show complete disappearance of GP both in the absence and presence of quenchers. **b.** Degradation efficiency of Sc-TBAPy towards 1.5 mM GP solution using ROS scavengers, showing 100 % degradation efficiency with no trace of GP in the solution.

Supplementary Table 5. ICP-OES results of Sc^{3+} leaking test on post GP degradation solution with Sc-TBAPy MOF.

Sc^{3+} concentration	Internal errors	Sc^{3+} degradation percentage of Sc- TBAPy MOF
1.58 ppm	0.05 ppm	0.42 %

Internal errors, including errors from standard solutions preparation and sample preparations. It is calculated by the following equation:

$$\text{Internal errors} = \sqrt[2]{Standard\ deviation\ of\ standard\ solutions^2 + Standard\ deviation\ of\ sample\ measurement\ results^2}$$

Sc^{3+} concentration of post GP degradation solution with Sc-TBAPy MOF is calculated from ICP-OES reading (632.75 ppb) multiplies by the dilution factor (2.5).

Sc^{3+} degradation percentage of Sc- TBAPy MOF was calculated by the following equation:

$$\%_{\text{Sc}^{3+}\text{ degradation}} = \frac{Sc^{3+}\ concentraion\ in\ post\ \text{ GP degradation solution}}{\text{Theoretical } Sc^{3+} \text{ degradation concentration}} \times 100\%$$

Where theoretical Sc^{3+} degradation concentration of Sc- TBAPy MOF was calculated by assuming 10 mg of Sc-TBAPy MOF were 100% degraded in 3 mL solution. 10 mg (1.25×10^{-5} moles) of Sc-TBAPy MOF, with formula of $[Sc_2(OH)_2(TBAPy)]$, obtains 2.49×10^{-5} moles of Sc^{3+}. When 10 mg of Sc-TBAPy MOF fully degraded in 3 mL solution, the concentration of Sc^{3+} of this solution is 1.12 mg of Sc^{3+} per 3 mL solution, which is 373.75 ppm (mg/L).

Supplementary Table 6. Comparison of Sc-TBAPy with other catalysts for glyphosate treatment.

Treatment process	Reaction Conditions	GP concentration (ppm)	GP conversion (%)	Ref
Sc-TBAPy/UV	pH 3.4, 10.7<br>reaction time: 8 h	253.6	100	This work
Al-TBAPy/UV	pH 3.4,<br>reaction time: 8 h	253.6	98 .33	This work
m-CBMOF-2/UV	reaction time<br>9 hr	1.69×10^3	70.4	[54]
m-CBMOF-2/UV	reaction time<br>9 h	3.4×10^3	82.8	[54]
NU-1000/UV	reaction time<br>9 h	3.4×10^3	95.6	[54]
TiO_2/UV	pH:3.5, reaction time: 30 min	42	92	[55]
Anatase TiO_2/UV	pH < 4, reaction time: 8 h	845.35	96	[56]
Rutile TiO_2 nanotubes/(modified with sulfuric acid)/UV	pH 7, O2<br>reaction time:<br>1 h	16.6	80	[57]
MnO_x-Al_2O_3/UV	pH 3, reaction time 30 min	1000	60	[58]
H_2O_2/UV	pH 3-10, reaction time: 8 h	30	71	[59]
O_3/H_2O_2	O_3, H_2O_2,<br>reaction time:15 min	0.00259 – 0.00365	99	[55]
Photo-Fenton	pH 2.8, Fe^{2+}/Fe^{3+}, H_2O_2,<br>reaction time: 2 h	100	100	[60]
Ozonation	pH 7, O_3<br>reaction time 30 min	42.3	100	[61]
Oxidative degradation	pH 5, birnessite, reaction time 100 h	100	55	[62]
Magnetite/UV	pH 7, reaction time 2 h	10	74	[63]
Geotite/UV	pH 7, reaction time 2 h	10	41	[63]
$HClO_4$/$NaClO_4$	pH 7, reaction time: 24 h	16.9	100	[64]
Electrochemical oxidation	pH 3-10, Anode: Ti/PbO_2,<br>reaction time:<br>6 h	4.3-33.8	95.5	[65]
Biodegradation	pH-7.4, bacteria, reaction time 104 h	1,500	90	[66]

Supplementary References:

1 Carey, P. H. *et al.* Valence and conduction band offsets in AZO/Ga2O3 heterostructures. *Vacuum* **141**, 103-108, doi:https://doi.org/10.1016/j.vacuum.2017.03.031 (2017).

2 Huang, R. *et al.* Angular dependent XPS study of surface band bending on Ga-polar n-GaN. *Applied Surface Science* **440**, 637-642, doi:https://doi.org/10.1016/j.apsusc.2018.01.196 (2018).

3 Krueger, T. D. *et al.* Illuminating Excited-State Intramolecular Proton Transfer of a Fungi-Derived Red Pigment for Sustainable Functional Materials. *The Journal of Physical Chemistry C* **126**, 459-477, doi:10.1021/acs.jpcc.1c09773 (2022).

4 Liu, W. *et al.* Panoramic portrait of primary molecular events preceding excited state proton transfer in water. *Chemical Science* **7**, 5484-5494, doi:10.1039/C6SC00672H (2016).

5 Chiu, N.-C. *et al.* Designing Dual-Functional Metal–Organic Frameworks for Photocatalysis. *Chem. Mater.* **34**, 8798-8807, doi:10.1021/acs.chemmater.2c02089 (2022).

6 de Halleux, V. *et al.* 1,3,6,8-Tetraphenylpyrene Derivatives: Towards Fluorescent Liquid-Crystalline Columns? *Adv. Funct. Mater.* **14**, 649-659, doi:https://doi.org/10.1002/adfm.200400006 (2004).

7 Oyamada, T. *et al.* Unusual photoluminescence characteristics of tetraphenylpyrene (TPPy) in various aggregated morphologies. *Chem. Phys. Lett.* **421**, 295-299, doi:https://doi.org/10.1016/j.cplett.2005.12.102 (2006).

8 Krueger, T. D. *et al.* Ultrafast Dynamics and Photoresponse of a Fungi-Derived Pigment Xylindein from Solution to Thin Films. *Chem. Eur. J.* **27**, 5627-5631, doi:https://doi.org/10.1002/chem.202005155 (2021).

9 Coleman, C. N. *et al.* Structural modulation of the photophysical and electronic properties of pyrene-based 3D metal–organic frameworks derived from s-block metals. *CrystEngComm* **23**, 82-90, doi:10.1039/D0CE01505A (2021).

10 Naito, K., Inada, Y. & Yamao, T. Optical and charge transport properties of 1,3,6,8-tetrakis(4′-methoxycarbonylphenyl)pyrene crystal. *Synth. Met.* **287**, 117086, doi:https://doi.org/10.1016/j.synthmet.2022.117086 (2022).

11 Wang, X. *et al.* Intermolecular Hydrogen-Bond-Assisted Solid-State Dual-Emission Molecules with Mechanical Force-Induced Enhanced Emission. *J. Org. Chem.* **87**, 8503-8514, doi:10.1021/acs.joc.2c00617 (2022).

12 Fang, C., Tang, L. & Chen, C. Unveiling Coupled Electronic and Vibrational Motions of Chromophores in Condensed Phases. *J. Chem. Phys.* **151**, 200901, doi:10.1063/1.5128388 (2019).

13 Brown, K. E., Salamant, W. A., Shoer, L. E., Young, R. M. & Wasielewski, M. R. Direct Observation of Ultrafast Excimer Formation in Covalent Perylenediimide Dimers Using Near-Infrared Transient Absorption Spectroscopy. *J. Phys. Chem. Lett.* **5**, 2588-2593, doi:10.1021/jz5011797 (2014).

14 Pensack, R. D., Ashmore, R. J., Paoletta, A. L. & Scholes, G. D. The Nature of Excimer Formation in Crystalline Pyrene Nanoparticles. *J. Phys. Chem. C* **122**, 21004-21017,

doi:10.1021/acs.jpcc.8b03963 (2018).
15 Yu, J., Park, J., Van Wyk, A., Rumbles, G. & Deria, P. Excited-State Electronic Properties in Zr-Based Metal–Organic Frameworks as a Function of a Topological Network. *J. Am. Chem. Soc.* **140**, 10488-10496, doi:10.1021/jacs.8b04980 (2018).
16 Ramakrishna, G., Bhaskar, A. & Goodson, T. Ultrafast Excited State Relaxation Dynamics of Branched Donor-π-Acceptor Chromophore: Evidence of a Charge-Delocalized State. *J. Phys. Chem. B* **110**, 20872-20878, doi:10.1021/jp063262h (2006).
17 Basuroy, K. *et al.* Ultrafast sorting: Excimeric π–π stacking distinguishes pyrene-N-methylacetamide isomers on the ultrafast time scale. *J. Chem. Phys.* **155**, 234304, doi:10.1063/5.0072785 (2021).
18 Deria, P., Yu, J., Smith, T. & Balaraman, R. P. Ground-State versus Excited-State Interchromophoric Interaction: Topology Dependent Excimer Contribution in Metal–Organic Framework Photophysics. *J. Am. Chem. Soc.* **139**, 5973-5983, doi:10.1021/jacs.7b02188 (2017).
19 Van Wyk, A., Smith, T., Park, J. & Deria, P. Charge-Transfer within Zr-Based Metal–Organic Framework: The Role of Polar Node. *J. Am. Chem. Soc.* **140**, 2756-2760, doi:10.1021/jacs.7b13211 (2018).
20 Stylianou, K. C. *et al.* A Guest-Responsive Fluorescent 3D Microporous Metal−Organic Framework Derived from a Long-Lifetime Pyrene Core. *J. Am. Chem. Soc.* **132**, 4119-4130, doi:10.1021/ja906041f (2010).
21 van Stokkum, I. H. M., Larsen, D. S. & van Grondelle, R. Global and Target Analysis of Time-Resolved Spectra. *Biochim. Biophys. Acta* **1657**, 82-104, doi:http://doi.org/10.1016/j.bbabio.2004.04.011 (2004).
22 Snellenburg, J. J., Laptenok, S. P., Seger, R., Mullen, K. M. & van Stokkum, I. H. M. Glotaran: A Java-Based Graphical User Interface for the R-Package TIMP. *J. Stat. Softw.* **49**, 1-22, doi:10.18637/jss.v049.i03 (2012).
23 Krueger, T. D. *et al.* Ultrafast Triplet State Formation in a Methylated Fungi-Derived Pigment: Toward Rational Molecular Design for Sustainable Optoelectronics. *J. Phys. Chem. C* **125**, 17565–17572, doi:10.1021/acs.jpcc.1c06260 (2021).
24 Kramar, B. V. *et al.* Single-Atom Metal Oxide Sites as Traps for Charge Separation in the Zirconium-Based Metal–Organic Framework NDC–NU-1000. *Energy Fuels* **35**, 19081-19095, doi:10.1021/acs.energyfuels.1c02623 (2021).
25 Liu, W. *et al.* Panoramic Portrait of Primary Molecular Events Preceding Excited State Proton Transfer in Water. *Chem. Sci.* **7**, 5484-5494, doi:10.1039/C6SC00672H (2016).
26 Gutierrez, M., Cohen, B., Sánchez, F. & Douhal, A. Photochemistry of Zr-based MOFs: ligand-to-cluster charge transfer, energy transfer and excimer formation, what else is there? *Phys. Chem. Chem. Phys.* **18**, 27761-27774, doi:10.1039/C6CP03791G (2016).
27 Chiu, N.-C. *et al.* Designing Dual-Functional Metal–Organic Frameworks for Photocatalysis. *Chem. Mater.* **34**, 8798–8807, doi:10.1021/acs.chemmater.2c02089 (2022).
28 Sung, J., Kim, P., Lee, Y. O., Kim, J. S. & Kim, D. Characterization of Ultrafast

Intramolecular Charge Transfer Dynamics in Pyrenyl Derivatives: Systematic Change of the Number of Peripheral *N*,*N*-Dimethyaniline Substituents. *J. Phys. Chem. Lett.* **2**, 818-823, doi:10.1021/jz200042s (2011).

29 Lee, S. & Kim, D. Symmetry-Dependent Intramolecular Charge Transfer Dynamics of Pyrene Derivatives Investigated by Two-Photon Excitation. *J. Phys. Chem. A* **120**, 9217-9223, doi:10.1021/acs.jpca.6b10819 (2016).

30 Niu, R. *et al.* D–π–A-Type Pyrene Derivatives with Different Push–Pull Properties: Broadband Absorption Response and Transient Dynamic Analysis. *J. Phys. Chem. C* **124**, 5345-5352, doi:10.1021/acs.jpcc.9b11667 (2020).

31 Vyas, V. S., Lindeman, S. V. & Rathore, R. Photophysical properties of 1,3,6,8-tetraarylpyrenes and their cation radicals. *J. Photochem. Photobiol. A* **375**, 209-218, doi:https://doi.org/10.1016/j.jphotochem.2019.01.014 (2019).

32 Sasaki, S., Drummen, G. P. C. & Konishi, G.-i. Recent Advances in Twisted Intramolecular Charge Transfer (TICT) Fluorescence and Related Phenomena in Materials Chemistry. *J. Mater. Chem. C* **4**, 2731-2743, doi:10.1039/C5TC03933A (2016).

33 Chen, C. & Fang, C. Fluorescence Modulation by Amines: Mechanistic Insights into Twisted Intramolecular Charge Transfer (TICT) and Beyond. *Chemosensors* **11**, 87, doi:10.3390/chemosensors11020087 (2023).

34 Tang, L., Wang, Y., Zhu, L., Lee, C. & Fang, C. Correlated Molecular Structural Motions for Photoprotection After Deep-UV Irradiation. *J. Phys. Chem. Lett.* **9**, 2311-2319, doi:10.1021/acs.jpclett.8b00999 (2018).

35 Kumpulainen, T., Lang, B., Rosspeintner, A. & Vauthey, E. Ultrafast Elementary Photochemical Processes of Organic Molecules in Liquid Solution. *Chem. Rev.* **117**, 10826-10939, doi:10.1021/acs.chemrev.6b00491 (2017).

36 Menšík, M., Rais, D., Pfleger, J. & Toman, P. Evolution of Diffusion Coefficient of Photoexcited Species in Excimer Forming Organic Thin Films. *J. Phys. Chem. C* **124**, 52-59, doi:10.1021/acs.jpcc.9b08953 (2020).

37 Malz, F. & Jancke, H. Validation of quantitative NMR. *Journal of Pharmaceutical and Biomedical Analysis* **38**, 813-823, doi:https://doi.org/10.1016/j.jpba.2005.01.043 (2005).

38 Bharti, S. K. & Roy, R. Quantitative 1H NMR spectroscopy. *TrAC Trends in Analytical Chemistry* **35**, 5-26, doi:https://doi.org/10.1016/j.trac.2012.02.007 (2012).

39 Kresse, G. & Hafner, J. Ab initio molecular dynamics for liquid metals. *Physical Review B* **47**, 558-561, doi:10.1103/PhysRevB.47.558 (1993).

40 Kresse, G. & Furthmüller, J. Efficiency of ab-initio total energy calculations for metals and semiconductors using a plane-wave basis set. *Computational Materials Science* **6**, 15-50, doi:https://doi.org/10.1016/0927-0256(96)00008-0 (1996).

41 Kresse, G. & Furthmüller, J. Efficient iterative schemes for ab initio total-energy calculations using a plane-wave basis set. *Physical Review B* **54**, 11169-11186, doi:10.1103/PhysRevB.54.11169 (1996).

42 Kresse, G. & Joubert, D. From ultrasoft pseudopotentials to the projector augmented-wave

method. *Physical Review B* **59**, 1758-1775, doi:10.1103/PhysRevB.59.1758 (1999).
43 Dudarev, S. L., Botton, G. A., Savrasov, S. Y., Humphreys, C. J. & Sutton, A. P. Electron-energy-loss spectra and the structural stability of nickel oxide: An LSDA+U study. *Physical Review B* **57**, 1505-1509, doi:10.1103/PhysRevB.57.1505 (1998).
44 Wang, V. X., N.; Liu, J. C.; Tang, G.; Geng, W.-T. VASPKIT: A User-Friendly Interface Facilitating High-Throughput Computing and Analysis Using VASP Code. *Comput. Phys. Commun.* **267**, doi:https://doi.org/10.1016/j.cpc.2021.108033 (2021).
45 VandeVondele, J. *et al.* Quickstep: Fast and accurate density functional calculations using a mixed Gaussian and plane waves approach. *Comput. Phys. Commun.* **167**, 103-128, doi:http://dx.doi.org/10.1016/j.cpc.2004.12.014 (2005).
46 Goedecker, S., Teter, M. & Hutter, J. Separable Dual-Space Gaussian Pseudopotentials. *Phys. Rev. B* **54**, 1703-1710, doi:10.1103/PhysRevB.54.1703 (1996).
47 Hartwigsen, C., Goedecker, S. & Hutter, J. Relativistic Separable Dual-Space Gaussian Pseudopotentials from H to Rn. *Phys. Rev. B* **58**, 3641-3662, doi:10.1103/PhysRevB.58.3641 (1998).
48 Krack, M. & Parrinello, M. All-electron ab-initio Molecular Dynamics. *Phys. Chem. Chem. Phys.* **2**, 2105-2112, doi:10.1039/b001167n (2000).
49 VandeVondele, J. & Hutter, J. Gaussian Basis Sets for Accurate Calculations on Molecular Systems in Gas and Condensed Phases. *J. Chem. Phys.* **127**, 114105, doi:10.1063/1.2770708 (2007).
50 Perdew, J. P., Burke, K. & Ernzerhof, M. Generalized gradient approximation made simple. *Phys. Rev. Lett.* **77**, 3865 (1996).
51 Grimme, S., Antony, J., Ehrlich, S. & Krieg, H. A Consistent and Accurate ab initio Parametrization of Density Functional Dispersion Correction (DFT-D) for the 94 Elements H-Pu. *J. Chem. Phys.* **132**, 154104, doi:10.1063/1.3382344 (2010).
52 Mandeep, Gulati, A. & Kakkar, R. DFT study of adsorption of glyphosate pesticide on Pt-Cu decorated pyridine-like nitrogen-doped graphene. *Journal of Nanoparticle Research* **22**, 17, doi:10.1007/s11051-019-4730-z (2020).
53 Zeng, X. *et al.* Simultaneously Tuning Charge Separation and Oxygen Reduction Pathway on Graphitic Carbon Nitride by Polyethylenimine for Boosted Photocatalytic Hydrogen Peroxide Production. *ACS Catalysis* **10**, 3697-3706, doi:10.1021/acscatal.9b05247 (2020).
54 Gan, L. *et al.* Biomimetic Photodegradation of Glyphosate in Carborane-Functionalized Nanoconfined Spaces. *J. Am. Chem. Soc.* **145**, 13730-13741, doi:10.1021/jacs.3c02019 (2023).
55 Chen, S. & Liu, Y. Study on the photocatalytic degradation of glyphosate by TiO2 photocatalyst. *Chemosphere* **67**, 1010-1017 (2007).
56 Musa, E. N. *et al.* Two Birds, One Stone: Coupling Hydrogen Production with Herbicide Degradation over Metal–Organic Framework-Derived Titanium Dioxide. *ACS Catal.* **13**, 3710-3722 (2023).
57 Zhang, G.-W. *et al.* Enhanced photocatalytic performance of titania nanotubes modified

with sulfuric acid. *Journal of Molecular Catalysis A: Chemical* **363-364**, 423-429, doi:https://doi.org/10.1016/j.molcata.2012.07.020 (2012).
58 Zheng, T., Sun, Y., Lin, Y., Wang, N. & Wang, P. Study on preparation of microwave absorbing MnOx/Al2O3 adsorbent and degradation of adsorbed glyphosate in MW–UV system. *Chemical Engineering Journal* **298**, 68-74, doi:https://doi.org/10.1016/j.cej.2016.03.143 (2016).
59 López, A., Coll, A., Lescano, M. & Zalazar, C. Advanced oxidation of commercial herbicides mixture: experimental design and phytotoxicity evaluation. *Environ. Sci. Poll. Res.* **25**, 21393-21402 (2018).
60 Souza, D. R. d., Trovó, A. G., Antoniosi Filho, N. R., Silva, M. A. & Machado, A. E. Degradation of the commercial herbicide glyphosate by photo-fenton process: evaluation of kinetic parameters and toxicity. *J. Braz. Chem. Soc.* **24**, 1451-1460 (2013).
61 Assalin, M. R., De Moraes, S. G., Queiroz, S. C., Ferracini, V. L. & Duran, N. Studies on degradation of glyphosate by several oxidative chemical processes: Ozonation, photolysis and heterogeneous photocatalysis. *J. Environ. Sci. Health* **45**, 89-94 (2009).
62 Barrett, K. A. & McBride, M. B. Oxidative Degradation of Glyphosate and Aminomethylphosphonate by Manganese Oxide. *Environmental Science & Technology* **39**, 9223-9228, doi:10.1021/es051342d (2005).
63 Yang, Y., Deng, Q., Yan, W., Jing, C. & Zhang, Y. Comparative study of glyphosate removal on goethite and magnetite: Adsorption and photo-degradation. *Chemical Engineering Journal* **352**, 581-589, doi:https://doi.org/10.1016/j.cej.2018.07.058 (2018).
64 Brosillon, S., Wolbert, D., Lemasle, M., Roche, P. & Mehrsheikh, A. Chlorination kinetics of glyphosate and its by-products: Modeling approach. *Water Research* **40**, 2113-2124, doi:https://doi.org/10.1016/j.watres.2006.03.028 (2006).
65 Tran, N., Drogui, P., Doan, T. L., Le, T. S. & Nguyen, H. C. Electrochemical degradation and mineralization of glyphosate herbicide. *Environmental Technology* **38**, 2939-2948, doi:10.1080/09593330.2017.1284268 (2017).
66 Firdous, S., Iqbal, S. & Anwar, S. Optimization and modeling of glyphosate biodegradation by a novel Comamonas odontotermitis P2 through response surface methodology. *Pedosphere* **30**, 618-627, doi:https://doi.org/10.1016/S1002-0160(17)60381-3 (2020).
